# Development and characterization of a CRISPR/Cas9n-based multiplex genome editing system for *Bacillus subtilis*

**DOI:** 10.1186/s13068-019-1537-1

**Published:** 2019-09-27

**Authors:** Dingyu Liu, Can Huang, Jiaxin Guo, Peiji Zhang, Tao Chen, Zhiwen Wang, Xueming Zhao

**Affiliations:** Frontier Science Center for Synthetic Biology and Key Laboratory of Systems Bioengineering (Ministry of Education), SynBio Research Platform, Collaborative Innovation Center of Chemical Science and Engineering (Tianjin), Department of Biochemical Engineering, School of Chemical Engineering and Technology, Tianjin University, Tianjin, 300072 China

**Keywords:** CRISPR/Cas9n, Multiplex genome editing, Combinatorial metabolic engineering, Riboflavin, Nick repair mechanism

## Abstract

**Background:**

Metabolic engineering has expanded from a focus on designs requiring a small number of genetic modifications to increasingly complex designs driven by advances in multiplex genome editing technologies. However, simultaneously modulating multiple genes on the chromosome remains challenging in *Bacillus subtilis*. Thus, developing an efficient and convenient method for *B. subtilis* multiplex genome editing is imperative.

**Results:**

Here, we developed a CRISPR/Cas9n-based multiplex genome editing system for iterative genome editing in *B. subtilis*. This system enabled us to introduce various types of genomic modifications with more satisfying efficiency than using CRISPR/Cas9, especially in multiplex gene editing. Our system achieved at least 80% efficiency for 1–8 kb gene deletions, at least 90% efficiency for 1–2 kb gene insertions, near 100% efficiency for site-directed mutagenesis, 23.6% efficiency for large DNA fragment deletion and near 50% efficiency for three simultaneous point mutations. The efficiency for multiplex gene editing was further improved by regulating the nick repair mechanism mediated by *ligD* gene, which finally led to roughly 65% efficiency for introducing three point mutations on the chromosome. To demonstrate its potential, we applied our system to simultaneously fine-tune three genes in the riboflavin operon and significantly improved the production of riboflavin in a single cycle.

**Conclusions:**

We present not only the iterative CRISPR/Cas9n system for *B. subtilis* but also the highest efficiency for simultaneous modulation of multiple genes on the chromosome in *B. subtilis* reported to date. We anticipate this CRISPR/Cas9n mediated system to greatly enhance the optimization of diverse biological systems via metabolic engineering and synthetic biology.

**Electronic supplementary material:**

The online version of this article (10.1186/s13068-019-1537-1) contains supplementary material, which is available to authorized users.

## Background

Metabolic engineering for the production of chemicals, fuels, and pharmaceuticals typically requires extensive modulation of metabolic networks to enhance the productivity of the host strains [1, 2]. The expression levels of pathway genes play a key role in determining the production of metabolites in an organism [3]. The toxic intermediates may accumulate with imbalanced expression of pathway genes, which can significantly negatively affect cell growth [4]. The construction of balanced metabolic pathways is consequently one of the most important research subjects of metabolic engineering and synthetic biology. The metabolic engineering ‘toolbox’ has expanded from a focus on individual genetic modifications to increasingly complex designs that require the modulation of multiple genes throughout the metabolic network of the cell [5–7]. To support this increased engineering complexity, powerfully iterative genetic engineering tools are increasingly being favored by metabolic engineers.

*Bacillus subtilis*, which was granted GRAS (generally regarded as safe) status by the US Food and Drug Administration, has long been widely used for the production of enzymes, drug precursors, platform compounds, biofuels and biopolymers [8]. It readily secretes products into the extracellular medium and can metabolize nearly any carbon source, making it an attractive biomanufacturing platform [9]. While *B. subtilis* is an ideal organism for metabolic engineering applications, the development of genetic tools is lagging behind popular production hosts such as *Escherichia coli* and *Saccharomyces cerevisiae*, especially in multiplex genome editing. In *E. coli* and *S. cerevisiae*, efficient genome editing tools and a range of established multiplex pathway-optimization techniques (MAGE [10], YOGE [11], TRMR [12], RAGE [13], CREATE [14], CHAnGE [15], and so on) have been developed, which enabled the simultaneous modification of several loci, and greatly enhanced the ability to engineer complex pathways. Although a substantial collection of counter-selectable markers [16–18], or λ-Red phage mediated single-stranded DNA recombination [19] tools are available for engineering *B. subtilis*, these techniques requires time-consuming sequential transformation steps and are unable to achieve highly efficient complex gene editing. The current genetic engineering tools, therefore, still represent a bottleneck for multiple genes modulation in *B. subtilis*.

Recently, microbial genome editing techniques have progressed significantly due to the extensive research conducted on the CRISPR system (clustered regularly interspaced short palindromic repeats), derived from the RNA-guided immune systems found in many bacteria and archaea [1, 20]. The introduction of the CRISPR/Cas9 system not only eliminated the need for selection markers in genome editing but also dramatically increased the editing efficiency [21–24]. Current genome editing applications based on the type II-CRISPR/Cas9 system in bacteria are classified into two categories: Cas9-mediated genome editing and Cas9 nickase (Cas9n)-mediated genome editing [25]. SpCas9 and SpCas9n induce the integration of the repair template into the target locus at similar frequencies. However, compared to Cas9-mediated genome editing methods, Cas9n causes less damage and toxicity to the host [26]. At the same time, the single-strand nick created by Cas9n is highly suitable for repair and thus improves the genome manipulation efficiency [27, 28]. CRISPR/Cas9n assisted genome editing tools have recently been developed for a number of bacteria, including *E. coli* [29], *Lactobacillus reuteri* [30], *Clostridium* sp. [31] and *Bacillus licheniformis* [32].

In *B. subtilis*, the CRISPR/Cas9 methodology has been established to introduce gene insertions, deletions and replacements [33–35]. Furthermore, double-target editing was achieved and reached high efficiency through the optimization of various editing template parameters and PAM sites [34]. However, a complicated experimental procedure had to be developed to facilitate this technique. Moreover, multiplex site-editing (more than two sites) using CRISPR/Cas9 was not feasible due to the low recombination efficiency and toxicity of the multiple Cas9-induced DSBs (double-strand breaks). Thus, it is necessary to develop a simple technique to simultaneously modulate multiple genes on the chromosome with high efficiency.

In this research, we developed a CRISPR/Cas9n-mediated genome editing technique for *B. subtilis*. Compared to recently reported CRISPR/Cas9-based genome modification systems, the Cas9n-mediated editing technique showed higher efficiency for large genomic deletions and multiplex editing. Subsequently, CRISPR/Cas9n-mediated multiplex gene editing was further improved by inhibiting nicks re-ligation. As a demonstration, the CRISPR/Cas9n-mediated multiplex genome editing was applied to generate a combinatorial RBS library of the riboflavin operon, in which three genetic loci were simultaneously modulated to improve the strain’s riboflavin production capacity.

## Results and discussions

### Investigation of the CRISPR/Cas-induced nickase system in *B. subtilis*

Recently, Cas9n-mediated genome editing has been shown to be an efficient and precise tool in a number of bacteria [30–32, 36, 37]. The nickase is an effective tool to circumvent DSB-induced lethality and the repair mechanism allows the resulting nicks to trigger HDR (homology directed repair) with less toxicity for the host cells [27]. In contrast to the DSB induced by native Cas9, the single-strand nick created by Cas9 nickase is more suitable for repair and thus improves the genome manipulation efficiency [27, 28]. We, therefore, exchanged the native Cas9 of existing constructs with Cas9n for use in *B. subtilis*, a substitution that may lead to higher survival frequencies of transformed cells and better editing efficiency.

**Fig. 1:**
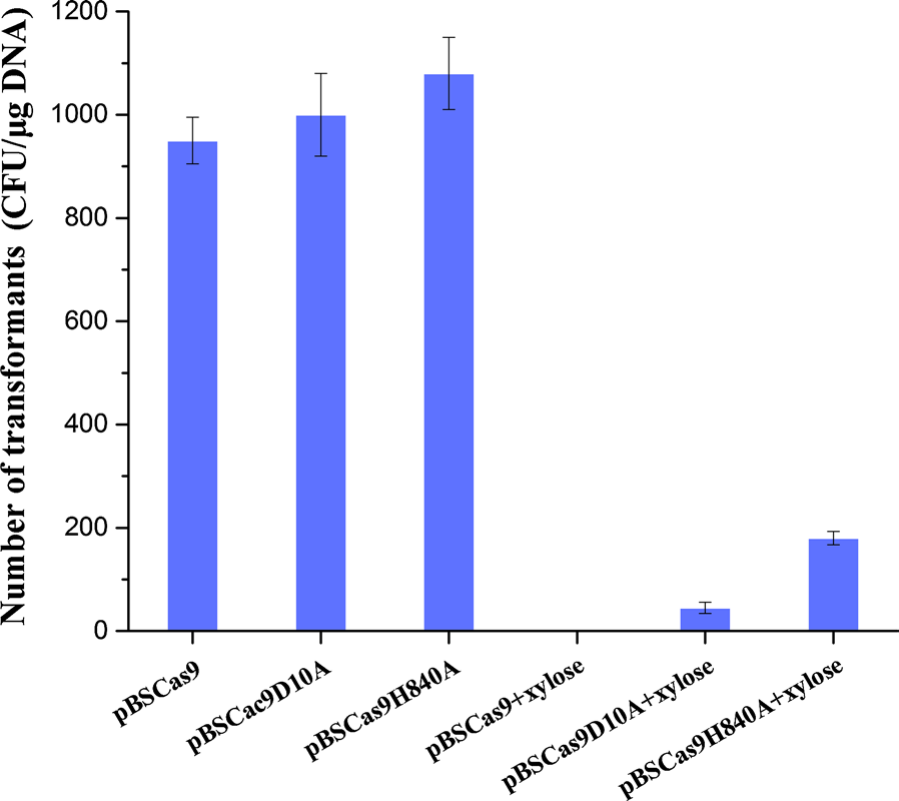
Transformation efficiency of different plasmids for CRISPR-Cas9/Cas9n-mediated gene editing. The target gene of the gRNA was the *amyE* locus. The error bars represent t standard deviations from three repeated experiments

To confirm that CRISPR/Cas9n was functional in *B. subtilis*, we tested different mutant versions of Cas9 without a repair template. In parallel, WT Cas9 was used as control. Cas9 and Cas9n (D10A and H840A) were individually introduced into *B. subtilis* together with co-expression of the target gRNA (*amyE*). The total colony forming units (CFU) were calculated by counting the corresponding transformants on agar plates (Fig. 1). The CFU number was reduced significantly due to the induced expression of Cas9/Cas9n, indicating that Cas9/Cas9n had most likely cleaved the genome as a consequence of the presence of the gRNA directed against *amyE*. By contrast, the transformation reached an appreciable frequency (~ 10^3^ CFU/µg DNA) when Cas9/Cas9n were not induced. These efficiencies of plasmid transformation were less than the average levels observed for *B. subtilis*, which may be due to leaky expression of Cas9/Cas9n from the induced promoter P*xylA*.

In this work, the transformation efficiencies with induced Cas9n were appreciably higher than with induced Cas9, and those of Cas9nH840A were higher than the efficiencies of Cas9nD10A. The transformation efficiency with induced Cas9nD10A was fourfold lower than that of Cas9nH840A (Fig. 1). These data indicated that Cas9n was functional in *B. subtilis* and Cas9nD10A had most likely cleaved the genome with higher efficiency than Cas9nH840A. Consequently, Cas9D10A nickase was employed in our CRISPR-Cas9n genome editing system, and Cas9n refers to the Cas9D10A mutant in further text.

### Establishment of CRISPR/Cas9n-mediated genome editing in *B. subtilis*

**Fig. 2:**
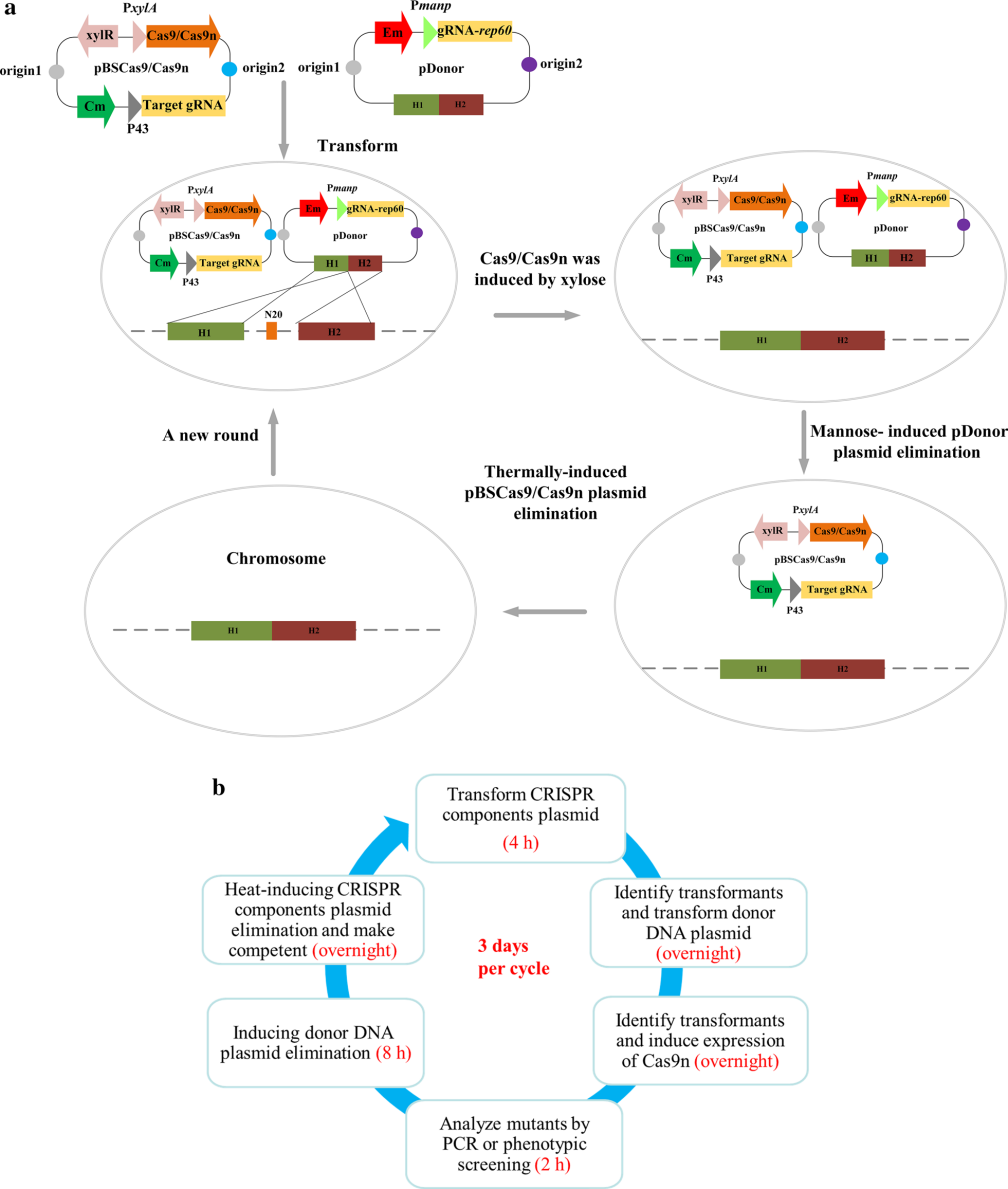
The CRISPR/Cas-mediated system for iterative genome editing. **a** The components and procedure of the CRISPR/Cas based system. Two plasmids respectively harboring gRNA and donor DNA are introduced into the cells, after which expression of Cas protein and homologous recombination are implemented. Gene modifications were introduced, allowing the cells to escape CRISPR mediated cleavage by abolishing the protospacer or the PAM sequences. When induced by mannose, gRNA targeting the *rep60* gene is expressed to eliminate the donor DNA plasmid. The gRNA plasmid was eliminated by inhibiting the replication of its thermo-sensitive replicon at increased temperature. **b** Step by step diagram of the iterative genome editing procedure. The time required for each step is shown in red

Recently, several CRISPR/Cas systems for *B. subtilis* have been developed using different design strategies, including single-plasmid systems [33, 38], two-plasmid systems [35] and chromosomal maintenance system [34]. Our CRISPR/Cas9n-mediated genome editing system is an improved two-plasmid system (Fig. 2a), which is composed of a vector encoding the CRISPR components and another vector carrying the donor DNA. The induced Cas9n expression system and gRNA constitutive expression cassettes were assembled on the CRISPR components plasmid (pBSCas9n), which also has a thermo-sensitive replication origin and a *cat* gene. The plasmids derived from pDonor, harboring the donor DNA, were designed to generate gene deletions, insertions, or replacements while altering the PAM sequences to allow mutant cells to escape CRISPR induced cell death. To construct the plasmid curing system, we used the mannose inducible promoter P*manp* to express a gRNA targeting the replication origin gene *rep60* on the donor DNA plasmid. For iterative genome editing, the *rep60*-targeting gRNA was expressed, which induced Cas9n to cleave the gRNA plasmid, resulting in plasmid elimination.

In the reported two plasmid systems of *B. subtilis*, gRNA and donor DNA were assembled in the same plasmid [35]. Our system separates the gRNA and donor DNA onto different vectors, which makes it easy and fast to complete the required plasmid construction, especially when assembling multiple gRNAs and donor DNAs. Compared to the single-plasmid and chromosomal maintenance systems, the two-plasmid system has the advantages of being suitable for complex modifications such as large fragment insertion and multiplex locus editing, as well as fast operation with high efficiency [39]. In this study, the optimized iterative editing system enjoyed the added advantage of modular construction protocols, reduced time consumption and increased convenience, which makes it more applicable to multiplex genome editing.

Each cycle of editing starts with the successive introduction of the plasmids pBSCas9n and pDonor into the cells. After inducing the expression of Cas9n, the hosts’ genome is cleaved by CRISPR-mediated digestion unless mutations were acquired at the PAM sequences. Spreading the induced cells on medium containing chloramphenicol and erythromycin allowed the selection of cells containing the desired modification. Subsequently, correct mutants were incubated for plasmid curing and then analyzed for antibiotic sensitivity to confirm the loss of the plasmid, after which single colonies were grown to prepare competent cells for the next round of editing. The time required for each editing cycle is 3 days (Fig. 2b).

### Characterization of CRISPR/Cas9n mediated genome editing in *B. subtilis*

The type II CRISPR-Cas9 system has been proven to be suitable for introducing various genome editing in *B. subtilis*, and was applied in the fields of metabolic engineering and synthetic biology [39]. Nevertheless, CRISPR/Cas9-mediated genome editing is still at the exploratory stage, especially because the necessary DSB caused by Cas9 may require longer incubation times for repair, or may even preclude the survival of cells after transformation with the CRISPR/Cas9 system. In this work, the alternative CRISPR/Cas9n-mediated genome editing system was established and characterized in *B. subtilis*, with the aim to overcome existing shortcomings of the classical CRISPR/Cas9 system. We, therefore, quantitatively compared and analyzed the effect of CRISPR/Cas9 and CRISPR/Cas9n on editing efficiency.

**Fig. 3:**
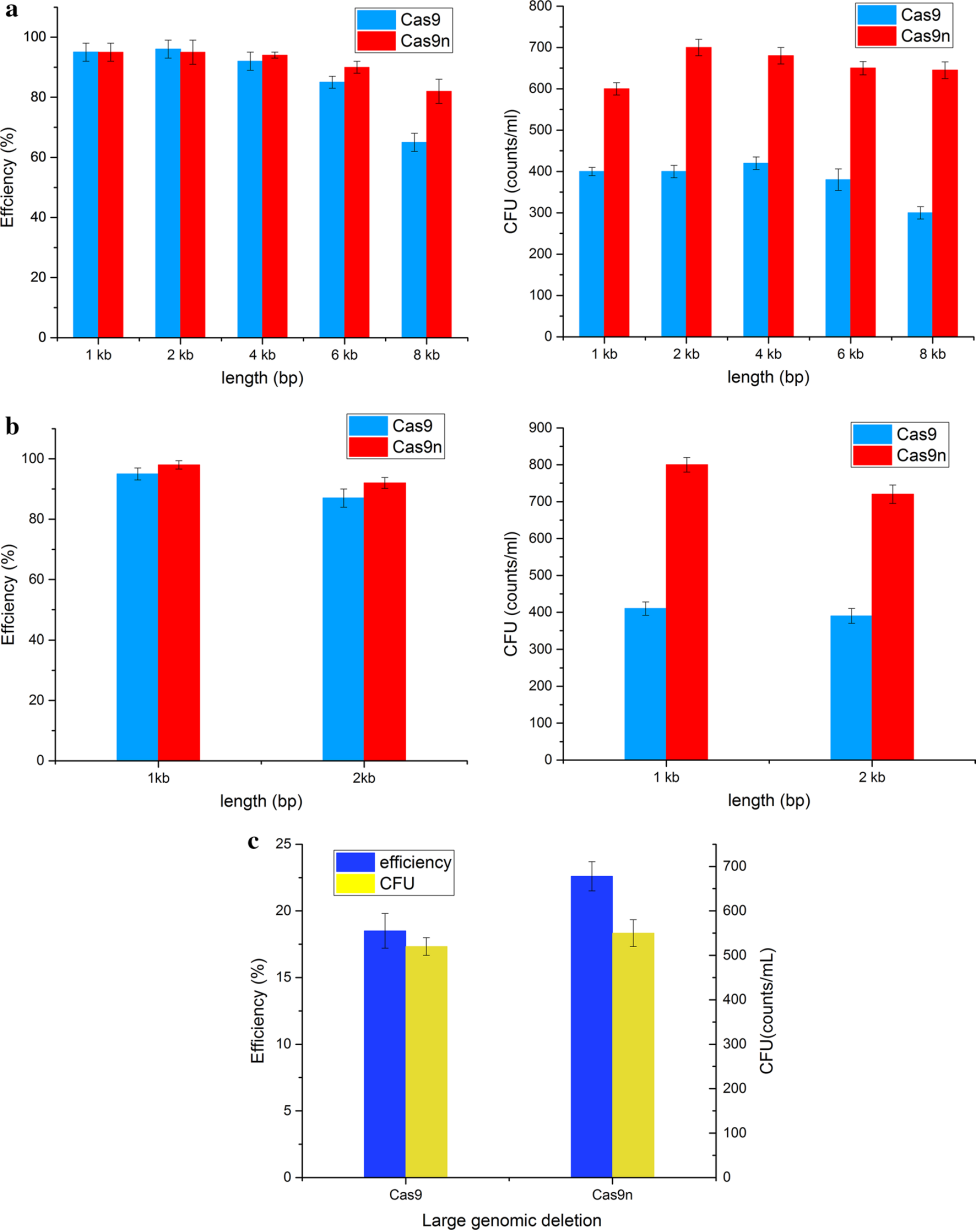
Characterization of CRISPR-Cas9/Cas9n mediated genome editing. **a** Editing efficiency and CFU for sequence deletions using CRISPR/Cas9 or CRISPR/Cas9n. For deletion, the region of the *amyE* gene and flanking sequences were deleted. **b** Editing efficiency and CFU for gene insertions. A deleted sequence in the *amyE* region was restored. **c** Editing efficiency and CFU for large genomic deletions. A prophage (-like) regions was deleted. In these genetic modifications, 500 bp homologous-arms were used for recombination. All error bars represent the value of standard deviation which were calculated from three repeated experiments

We first systematically evaluated the ability of CRISPR-Cas9/Cas9n mediated gene editing to introduce various types of modifications including gene deletions, insertions and deletions of large-scale chromosomal regions (Fig. 3). As shown in Fig. 3a, our system yielded at least 90% editing efficiency for 1–4 kb gene deletions with both CRISPR/Cas9 and CRISPR/Cas9n. However, the efficiency for 6 kb and 8 kb gene deletions with Cas9 as editing tool decreased dramatically in our system. By contrast, the efficiency with Cas9n remained above 80%. The CRISPR/Cas9n system produced 98% correct 1 kb gene insertions and 92% 2 kb gene insertions, which was better than the results obtained with CRISPR/Cas9 (95% and 87%, respectively) (Fig. 3b). Moreover, the ability of the CRISPR/Cas9n system to introduce large genomic deletions was slightly better than that of CRISPR/Cas9 (Fig. 3c). Large DNA fragment deletions remain problematic with the traditional genome editing method. Recent large genomic deletion studies have reported low mutation efficiency [40] or were only feasible in specific strains containing antibiotic resistance markers [41]. Recently, CRISPR/Cas9n-based dual-targeted nicks have been used to delete 25.1 kb fragment from the genome of *B. subtilis* with the efficiency of 53% [37]. In this study, a prophage (-like) regions, a large DNA fragment of 20.5 kb was completely deleted using CRISPR/Cas9n. Although the deletion efficiency for large fragment only reached 23.6% by our CRISPR/Cas9n system, only one nick was targeted in this study. This operation was simple and successful, and the efficiency was higher than our previous marker-free strategy [16].

In addition to the advantages in editing efficiency, the CFU number in almost all the experiments with CRISPR/Cas9n as editing tool were higher than with CRISPR/Cas9 (Fig. 3a–c), indicating a reduced toxicity of the former to the host cells in the process of genome editing. Hence, the CRISPR/Cas9n system was more fault-tolerant in practical molecular cloning operations.

To our best knowledge, the CRISPR system based on Cas9n-induced SSBs (single-strand breaks) has not been previously characterized systematically in *B. subtilis*. Hence, this is the comprehensive report of the successful use of such a system in *B. subtilis*. In our editing system, Cas9-mediated genome modification achieved a similar efficiency to previous reports of CRISPR/Cas9 system for *B. subtilis* [33–35, 38]. However, Cas9n causes less damage to the host than the CRISPR/Cas9 genome modification system and allows for more precise genome editing [36, 42]. Based on our results, the CRISPR/Cas9n system constructed in this study can be applied to 1–6 kb gene deletions and 1–2 kb gene insertions using fixed-length homology arms (500 bp) with at least 90% efficiency. Furthermore, the Cas9n-based system has the advantage of long fragment deletion with higher efficiency. This is because the single-strand nick created by Cas9n is prone to homologous recombination repair and thus improves the genome editing efficiency [27, 43]. In addition to the higher editing efficiency, more CFU could be obtained by genome editing with CRISPR/Cas9n. The positive clones are thus easier to obtain in practice, especially when editing genes associated with cell growth.

### Multiplex genome editing mediated by CRISPR/Cas9n system

**Fig. 4:**
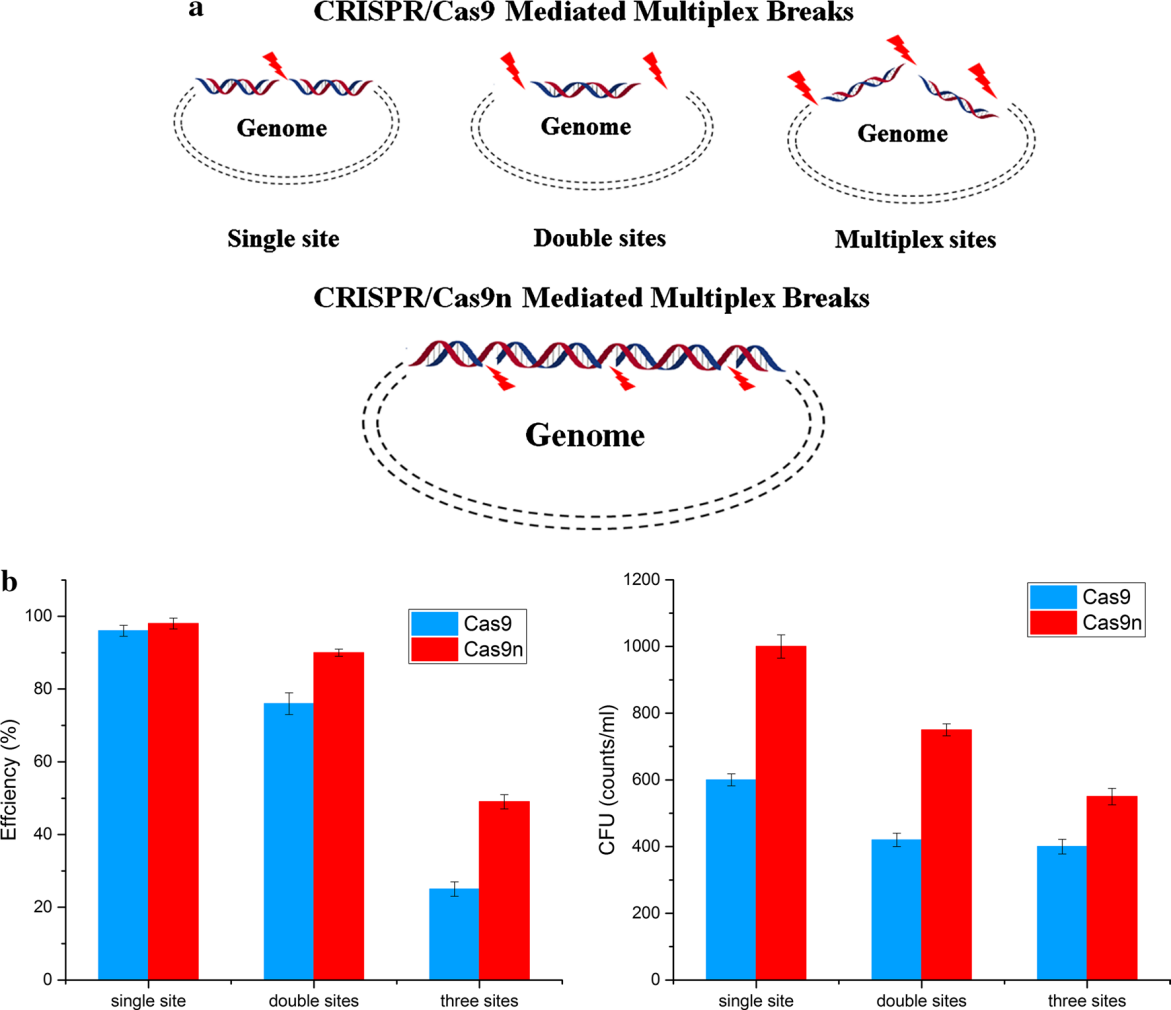
Strategy for CRISPR-Cas9/Cas9n mediated multiplex point mutations. **a** Assumed mechanism of CRISPR-Cas9/Cas9n mediated multiplex breaks. **b** Editing efficiency and CFU for multiplex point mutations using the CRISPR-Cas9/Cas9n system. In these genetic modifications, 500 bp homologous-arms were used to achieve recombination. All error bars represent the value of standard deviation which were caculated from three repeated experiments

The introduction of genomic point mutations is widely used strategy in metabolic engineering, including site-directed mutagenesis, RBS optimization, and so on. The recently developed CRISPR/Cas9 based point mutation systems for *B. subtilis* reached up to 100% editing efficiency for single-point mutations [35]. However, the efficiency for two simultaneous mutations decreased dramatically, and the modification of three sites was not attempted in *B. subtilis*, suggesting a limitation for combinatorial modulation. DSB based on Cas9 is a powerful counter-selection marker that ensures the high apparent editing rate among the obtained colonies. However, it is so strong that the cell survival rate decreases dramatically as the number of target locis increase. Multiple genomic cleavage sites based on Cas9 are more difficult to repair by HDR because more breaks and steric hindrance might arise with the increase in the number of target loci based on DSB (Fig. 4a). In contrast to the DSB based Cas9, nickase can facilitate homology-directed repair with minimal off-site mutagenic activity [26]. The genome is considered an integrated structure, although it can be converted into cyclic annular nicked DNA by the action of nickase (Fig. 4a). Nicked genomic DNA is typically repaired either seamlessly or through high-fidelity HDR, and it is easier to repair than cleaved genomic DNA containing DSB. Thus, we presumed that the CRISPR/Cas9n system enables more efficient introduction of multiplex point mutations for gene editing.

Based on this hypothesis, we explored the ability of our CRISPR-based system to introduce single and several simultaneous point mutations. We targeted three sites, *amyE*, *upp*, and *sigE*, and designed donor DNAs to introduce codon replacements in these target genes. The two plasmids harboring the corresponding gRNAs and donor DNAs were designed and constructed in a modular fashion. Two CRISPR systems were evaluated by introducing codon replacements in the PAM sequences. The results showed that single-point mutations could be introduced with near 100% efficiency using either CRISPR/Cas9 or CRISPR/Cas9n. However, increasing the number of target sites in the CRISPR/Cas9 system, significantly decreased the editing efficiency, with only 19.5% correct clones for three-point mutations. In contrast to CRISPR/Cas9, the CRISPR/Cas9n system maintained a more desirable editing efficiency for multiplex genome editing, with 90% efficiency for two mutations and 49% efficiency for three mutations (Fig. 4b).

We employed the CRISPR/Cas9n methodology to overcome some deficiencies of the DSB approach and introduce multiplex mutations. To our best knowledge, the 49% observed in this study is the highest efficiency for simultaneous modulation of multiple genes on the chromosome in *B. subtilis* reported to date. Recently, a CRISPR/Cas9-facilitated multiplex pathway optimization technique was developed in *E. coli*, by co-expressing λ-Red recombineering system and Cas9 system, which yielded 70% efficiency for modulating three target loci simultaneously [44]. However, a similar CRISPR/Cas9-medited system could not achieve satisfying efficiency for multiplex pathway modification in *B. subtilis*. We inferred that the low efficiency of multiplex mutations is due to the comparatively inefficient HDR without expressing an exogenous recombination system and the low cell survival rate due to the multiple Cas9-induced DSBs. Furthermore, the low plasmid transformation efficiency of *B. subtilis* compared to *E. coli* is also a reason for the low multiplexing efficiency. The determination of the positions of the PAM sequences and design of gRNAs greatly influenced the efficiency of CRISPR-based modulation [33, 45, 46]. The expression of an exogenous recombination system and optimization of gRNAs may further boost the frequency of homologous recombination and thus contribute to the efficiency of CRISPR/Cas9n mediated gene editing. In addition, regulation of the nick repair mechanism may improve CRISPR/Cas9n mediated genome editing.

### Improving CRISPR/Cas9n mediated multiplexing by inhibiting nicks re-ligation in *B. subtilis*

Although CRISPR/Cas9n-medited multiplex point mutations could be introduced with excellent efficiency, there was a certain number of false-positive colonies in the process of gene editing. Nicks are efficiently re-ligated and this would be predicted to compete with homologous recombination to reduce the efficiency of gene editing [26, 27]. We suspected that false-positive colonies arose from cells whose genome had not been cleaved and from cells that have been repaired by the non-HDR mechanism. Therefore, the non-HDR repair mechanism of Cas9n-induced SSB should be suppressed to improve the editing efficiency based on HDR repair.

**Fig. 5:**
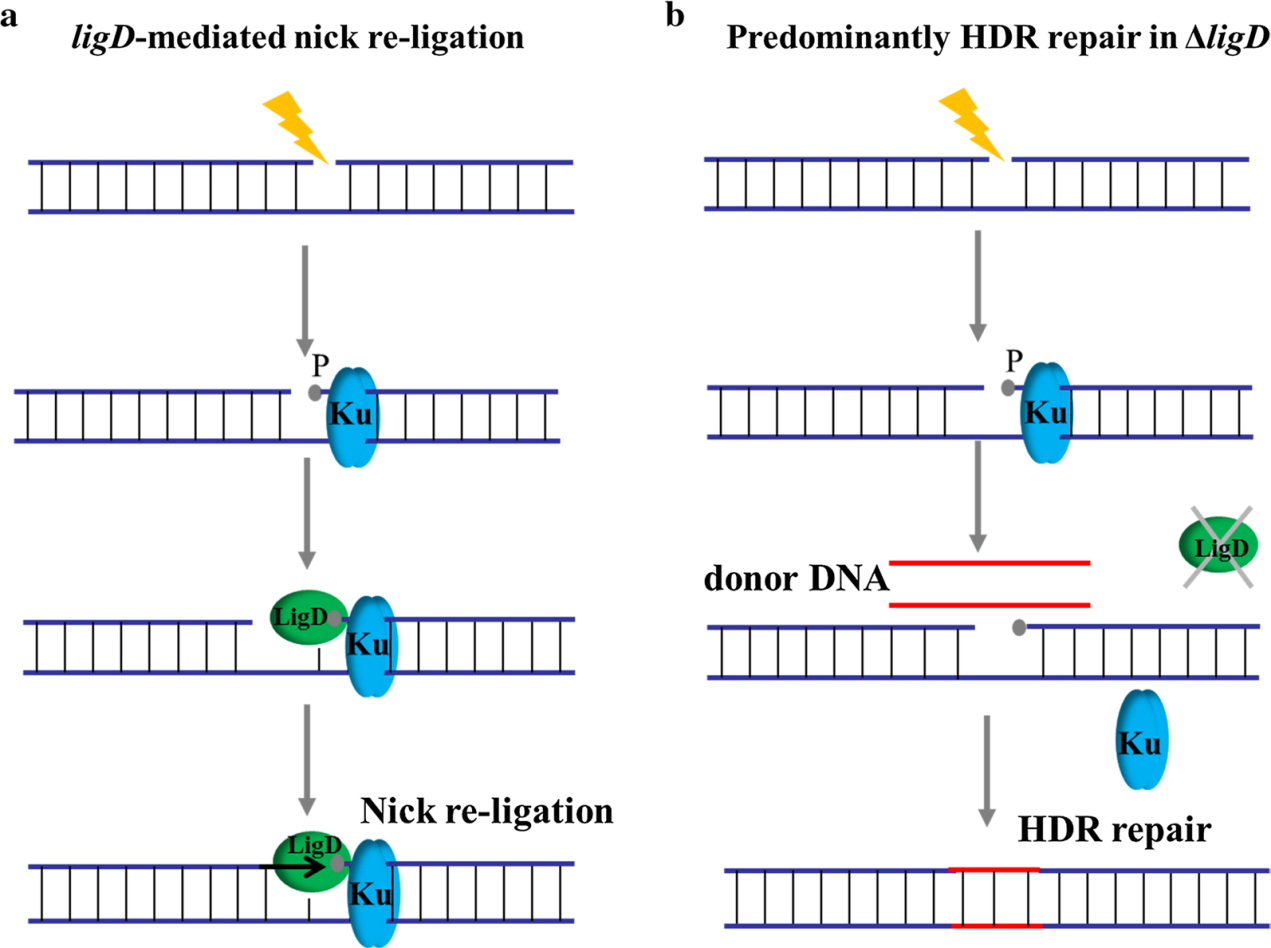
Mechanism of *ligD* mediated nick ligation in *B. subtilis*. **a** The *ligD*-mediated nick re-ligation. A Ku homodimer binds to the end of the DNA break and recruits LigD protein. The polymerase domain of LigD specifically binds to a 5′-phosphate (P) and promotes end-synapsis-, together with protein Ku. The nuclease and polymerase activities of LigD, and possibly other factors, process the break termini, if required, to restore complementary ends. Finally, ligation of the nick by LigD repairs the break. **b** Predominantly HDR repair in Δ*ligD*. When LigD is repressed, the DNA break is repaired by the HDR mechanism

The re-ligation of nicks depends on DNA ligases, including NAD^+^-dependent ligase (encoded by gene *ligA*) and ATP- dependent ligase (encoded by gene *ligD*) in *B. subtilis*. The gene *ligA* is challenging to engineer because it is an essential gene of *B. subtilis*, which is associated with DNA replication and repair. By contrast, the gene *ligD*, whose corresponding protein uses ATP to form a phosphodiester at the site of a SSB and catalyze DNA ligation [47], is an appropriate target in regulation of nicks re-ligation in *B. subtilis*. The underlying mechanism is similar to the NHEJ (non-homologous end joining) pathway, which is one of the major mechanisms for repairing strand breaks that occur in genomic DNA [48] (Fig. 5a). Thus, the *ligD* gene was targeted to regulate the SSB repair pathway. We presumed that knocking out *ligD* would further improve the efficiency of multiplex genome editing (Fig. 5b).

**Fig. 6:**
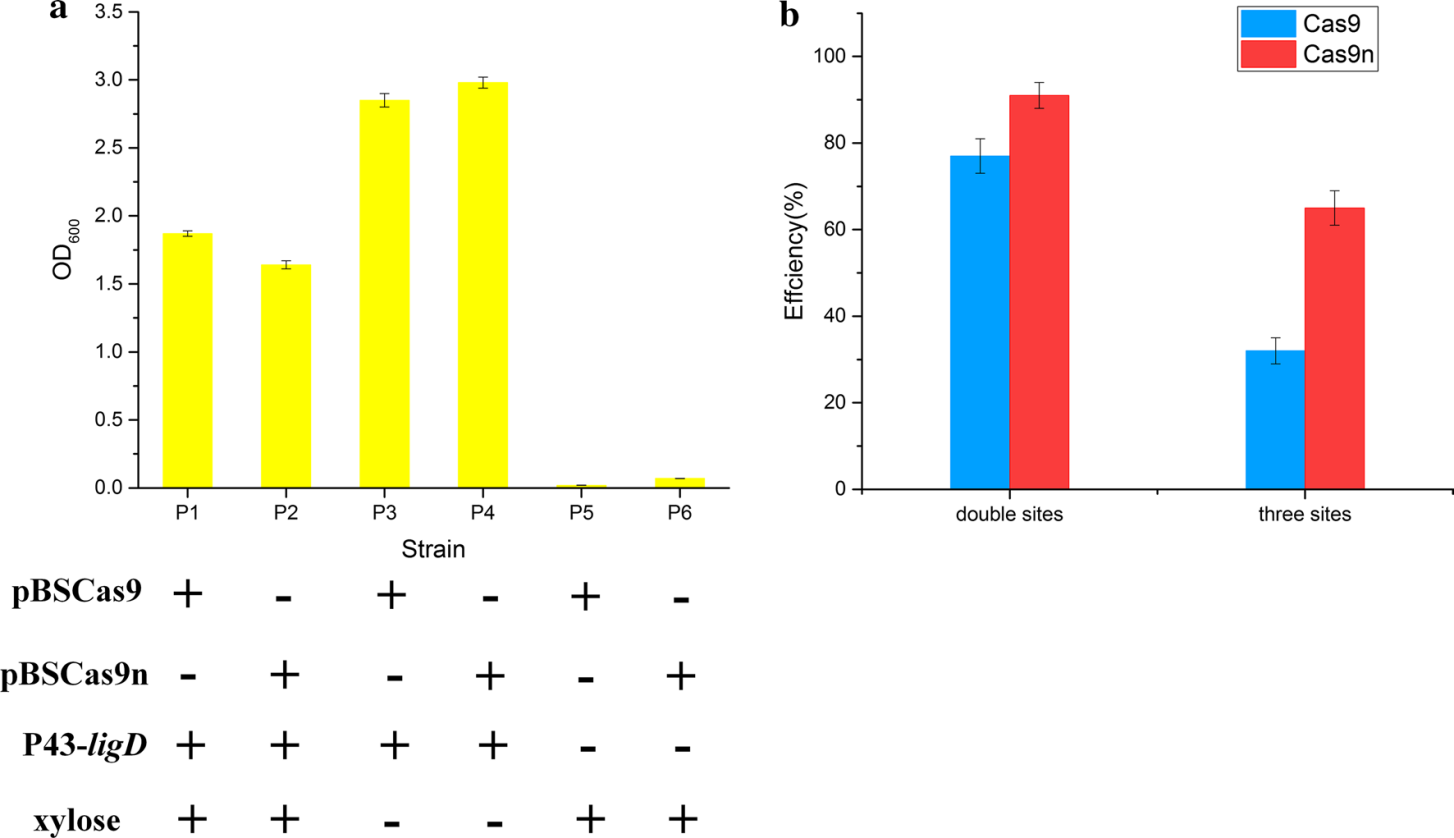
Strategy for improving CRISPR-Cas9/Cas9n mediated gene editing by regulating *ligD*. **a** Cell growths of strains with CRISPR-Cas9/Cas9n mediated gene editing under different induction conditions. Promoter P43 was used to overexpress *ligD*. The *amyE* was targeted for Cas9/Cas9n-induced genome cleavage in this study. **b** Editing efficiency for multiplex point mutations by the improved CRISPR-Cas9/Cas9n system. In these genetic modifications, 500 bp homologous-arms were used to achieve recombination. All error bars represent the value of standard deviation which were caculated from three repeated experiments

Firstly, the function of *ligD* was characterized and investigated in *B. subtilis* harboring CRISPR-Cas9/Cas9n system. It was overexpressed from plasmid pHP13 to repair Cas9/Cas9n-induced genome cleavage. As shown in Fig. 6a, cell growth of strains P5 and P6 was severely affected by the Cas9/Cas9n-induced genomic cleavage. By contrast, cell growth of strains P1 and P2 was dramatically improved by enhancing the repair of nicks via overexpression of *ligD*. Moreover, knockout of *ligD* in wild type *B. subtilis* 168 had no obvious influence with cell growth (Additional file 2: Figure S4). We next assessed the efficiency of CRISPR/Cas9n-induced multiplex point mutations in *B. subtilis* 168Δ*ligD*. The same sites as before were targeted to introduce codon replacements. As shown in Fig. 6b, CRISPR/Cas9n-mediated multiplex gene editing was further enhanced, reaching 91% efficiency for two-point mutations and 65% efficiency for three-point mutations. Thus, the improved CRISPR/Cas9n system achieved higher efficiency for the simultaneous modification of several loci, which enables the implementation of multiplex metabolic engineering on the chromosome of *B. subtilis*.

For the first time, we employed the *ligD* mediated repair mechanism to improve CRISPR/Cas9n mediated multiplex genome editing. The efficiency for simultaneous modulation of three sites was further increased to 65%. The *ligD* gene, which is involved in NHEJ repair and related pathways, is a stable expressed and nonessential in the *B. subtilis*. It should be noted that NHEJ does not work efficiently or is not present in most bacteria, but *Bacillus* species possess a conserved prokaryotic NHEJ pathway that is essential for repair of strand breaks arising in the stationary phase and spore dormancy period [49, 50]. In essence, the re-ligation of strand breaks is mediated by a two-component Ku-ligase break repair complex. The knockout of *ligD* facilitated the repair of HDR mediated strand breaks by inhibiting the nicks re-ligation mechanism (Fig. 5). This type of DNA ligase-based repair mechanism should be paid attention to the research of genome editing for *Bacillus* species.

### Application of CRISPR/Cas9n-mediated multiplex genome editing for combinatorial pathway modulation

To demonstrate the applicability of CRISPR/Cas9n-mediated multiplexing, genetic components of the *B. subtilis* riboflavin operon were modulated in a combinatorial fashion to optimize the metabolic efficiency of riboflavin biosynthesis. The plasmid library pDonor-ribRBSLib provided the donor DNAs for homologous recombination, encompassing three modulation libraries for the simultaneous regulation of the *ribB*, *ribA*, and *ribH* genes. The corresponding gRNA plasmid pBSCas9n-gRNArib, which functioned to target the Cas9n protein to the regulator sequences of *ribB*, *ribA* and *ribH*, was constructed to encode three gRNAs with their N20 sequences complementary to the native RBS regions of these genes.

**Fig. 7:**
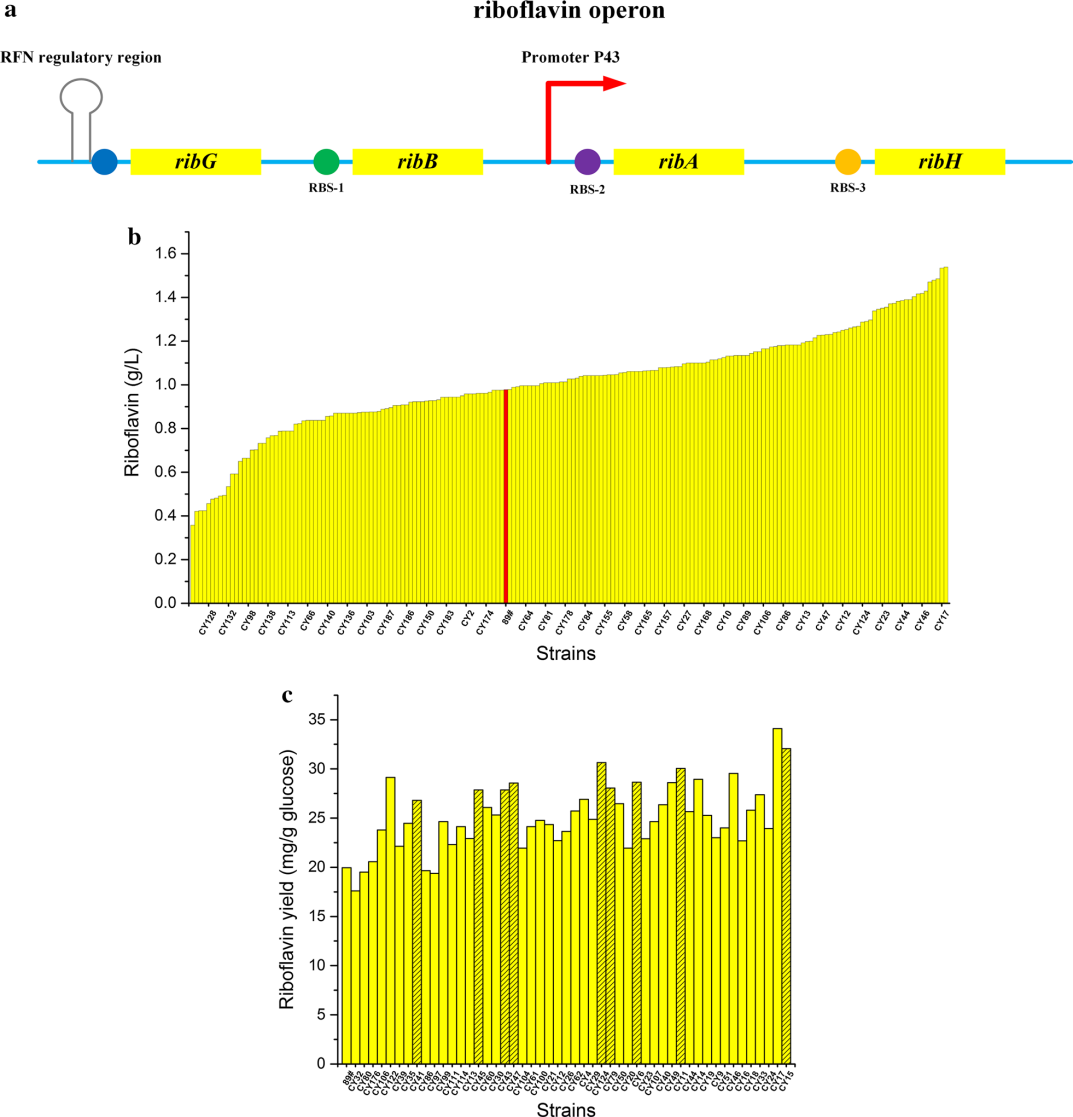
Optimization of the riboflavin operon by improved CRISPR/Cas9n mediated multiplex gene editing. **a** Integration of the riboflavin synthesis pathway genes and regulatory elements. **b** Production of riboflavin in 96-well plates. The red bar shows the control strain. **c** Yield of riboflavin in 96-well plates. Striped bar show strains that were selected for secondary screening due to high yields, which were not among the 20 highest-producing strains. The data show the average values and standard deviations of triplicate experiments

In *B. subtilis*, the riboflavin biosynthesis genes, *ribG*, *ribB*, *ribA* and *ribH*, are clustered in a single operon, the structure of which was studied thoroughly (Fig. 7a). In our previous study, strain BS89 was constructed for riboflavin production by deregulating *rib* operon via overexpression of *ribA* [51]. In this study, to further deregulate the *rib* operon, three genes (*ribB*, *ribA* and *ribH*) involved in riboflavin biosynthesis were engineered through combinatorial modulation of their RBS to balance and optimize their expression for improved riboflavin production.

To achieve the desired efficiency for multiple modulations, BS89 was engineered by deleting *ligD* to generate BS89Δ*ligD*. The plasmids pBSCas9n-gRNArib and pDonor-ribRBSLib were introduced into BS89Δ*ligD* to initiate homologous recombination to replace the native RBS regions of *ribB*, *ribA* and *ribH*. The *B. subtilis* library carrying two plasmids was incubated for 8 h in medium with 1% xylose. The cultured library was diluted and spread on LB agar plates containing appropriate antibiotics. The colonies were preliminarily assessed by visually screening yellow color. Nearly 200 yellow colonies were selected, and these strains were characterized in 96-well plate fermentations. Eventually, 190 strains were cultivated to produce riboflavin in the 96-well plate (Fig. 7b). Compared to BS89, 111 strains were improved in terms of riboflavin production. DNA sequencing was employed to analyze the 20 colonies with the highest production using the primer pairs P1-F/L, P2-F/L and P3-F/L. Based on the DNA sequence analysis, 11 colonies had modulations in all three genetic targets, while the other had two modulations genes. According to the DNA sequencing data, the multiplex library had good diversity for metabolic optimization. To analyze the production performance of the strains systematically and comprehensively, the yields of the 50 most productive strains were determined (Fig. 7c).

In this work, the efficiency for three-point mutations was about 50%, which was lower than the expected efficiency (65%). In practice, the multiplexing efficiency was limited by several genome editing factors, specifically the PAM site sensitivity and relative position of selected targets. It has been observed that certain PAM sites are less susceptible to CRISPR system-mediated strand breaks since the editing efficiency can vary substantially between PAM sites in a single gene [52]. The problem in gRNA design may be associated with the formation of potential secondary structures. Several secondary structures may reduce the binding capacity (or frequency of binding) of Cas9/Cas9n to the gRNA [53]. In this work, we did not implement optimization in term of PAM sites and gRNAs. The three target genes are arranged sequentially in the operon (SubtiList positions of *ribG*, *ribB ribA*: 2430.5 kb, 2429.4 kb, 2428.8 kb) (Fig. 7a) and the efficiency of homologous recombination may have decreased due to their close relative position compared to above-test genes (SubtiList positions of *amyE*, *upp sigE*: 327.2 kb, 3788.1 kb, 1604.1 kb). Nonetheless, the multiplexing efficiency still exceeded 50% in our sample. This indicated that the CRISPR/Cas9n system we developed and optimized had advantages for multiplex genome editing and made it possible to modulate multiple genes on the chromosome simultaneously in metabolic engineering of *B. subtilis*.

### Verification of the optimized strains and analysis of their RBS regions

**Fig. 8:**
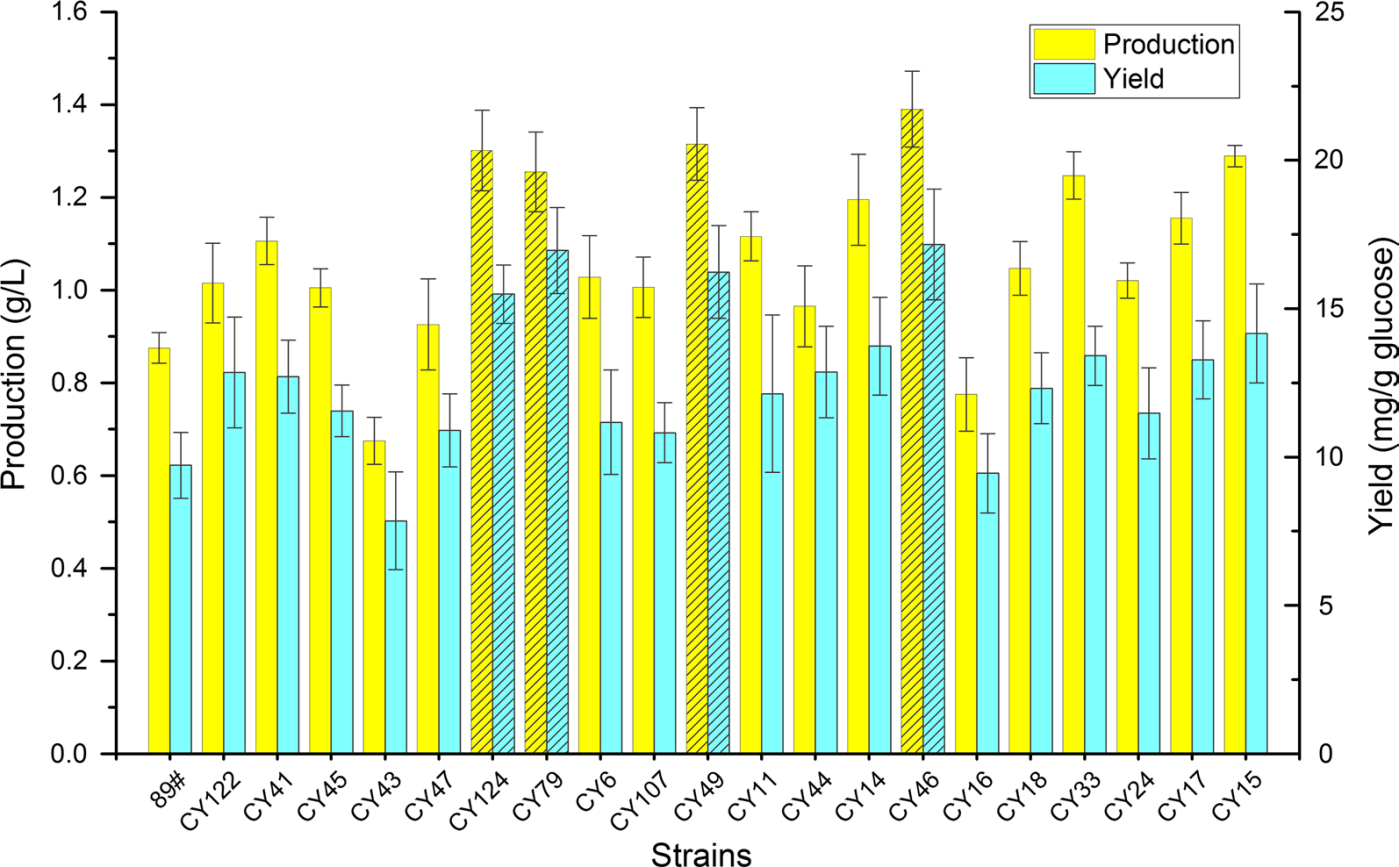
Productions and yields of riboflavin in shake-flask fermentation. The strains were cultivated aerobically in 50 mL of YE medium in 500 mL shake-flask at 240 rpm and 41 °C with an initial inoculum of 2% (v/v). Striped bar indicate strains for which the 5′-UTR regions of the mRNA secondary structure of riboflavin operon genes were simulated. The data are the average values and standard deviations from triplicate experiments

Based on the results of productions and yields, a total of 20 strains were chosen for verification in shake-flask fermentations. The 14 strains were chosen from the top 20 highest production strains, including 11 strains with three modulated genes (CY17, CY24, CY33, CY18, CY16, CY46, CY14, CY44, CY49, CY107 and CY23) and 3 strains with two modulated genes (CY15, CY11 and CY6). Besides the above 14 strains, another 6 high yield strains were chosen from the other 30 high production strains (CY41, CY45, CY43, CY47, CY124 and CY79). Compared to BS89, a total of 18 strains were improved in terms of riboflavin production (Fig. 8), and the best strain CY46 produced 1.39 g/L riboflavin with a yield of 17.16 mg/g glucose, which represents a 59% increase over the control strain BS89.

The RBS regions of the 20 optimized strains were determined by DNA sequencing (Table 1). In bacteria, RBS are effective control elements for translation initiation and thereby protein expression. Previous studies have generated libraries of RBS with the goal of optimizing the function of a genetic system [54, 55]. In our work, the riboflavin operon was optimized to improve riboflavin production. To investigate the reason for this phenomenon, the secondary structure in the 5′-UTR region of the mRNAs of riboflavin operon genes in the 4 highest production strains (CY124, CY79, CY49 and CY46) were simulated using the RNAfold webserver (http://rna.tbi.univie.ac.at/) with default settings. The RBS structures of the high production strains showed similar changes compared to those of strain BS89. The results indicated that the RBS of BS89-*ribA* is hidden in a hairpin structure (Additional file 2: Figure S5), while the RBS of CY46-*ribA* in the highest production strain CY46 is exposed (Additional file 2: Figure S6). The *ribA* is a key rate-limiting gene in riboflavin biosynthesis and has a positive direct impact on the production of riboflavin. The hairpin structure covered the RBS of BS89-*ribA* and impeded ribosome binding to the mRNA to start translation, which may explain the low efficiency of this construct. Furthermore, the RBS of BS89-*ribH* is located in a loop structure (Additional file 2: Figure S5), while the RBS of CY46-*ribH* is more exposed (Additional file 2: Figure S6). In contrast to the improved expression of *ribA* and *ribH*, the translational levels of *ribB* were decreased in the strain CY46. The RBS of BS89-*ribB* is more exposed than the RBS of CY46-*ribB* (Additional file 2: Figure S5 and Figure S6). This demonstrated that the optimized expression of the operon depends on the balanced expression of various genes rather than on overexpressing every gene monotonously. The corresponding RBS sequences of strains CY124, CY79 and CY49 mainly obeyed the described structure laws, except for the RBSs of CY79-*ribH* and CY124-*ribH* (Additional file 2: Figure S7, Figure S8 and Figure S9).

**Table 1:** Sequences in RBS regions of the riboflavin operon genes of the 20 optimized strains

Strain	RBS region of *ribB*	RBS region of *ribA*	RBS region of *ribH*
CY23	ATATAAG	GTAAGAA	AAGAAGG
CY41	ATTAGAG	AAGGGGA	AGTGAGA
CY45	**ATGGTGA** ^a^	AGGTGGA	**ATGGGAA**
CY43	AGACAGG	GGCTGGA	AGATGAG
CY47	AACATAG	**AAGGAGG**	ATAGAGA
CY124	AATGAGA	ACAAGGA	AGGATGG
CY79	AGTAAGA	AAAGGAG	GTAGGAA
CY6	**ATGGTGA**	---GAGG^b^	AGTAGAA
CY107	GGAGAGG	ATTGGAG	AAGAGGA
CY49	ATTGAGG	AGTCGAA	GTAAAAG
CY11	AGTCGAA	**AAGGAGG**	---GGAG
CY44	GGGAGAA	ATGTGAA	GGCTAGG
CY14	GGAGTAG	GATGGAA	AGTTGAG
CY46	AGTTGAG	AAAGAGA	AAGATAA
CY16	AATGGAG	AATGGAG	AGTTGAG
CY18	GCGAAGG	AGCGAGG	ATGGGAG
CY33	AGGGAGA	AATGGAG	GCGAAGG
CY24	GTTGAAG	AGGAGAA	AAGAAAG
CY17	AAAGAGG	ATTGGAG	AGCAGGA
CY15	AGGAGGA	**AAGGAGG**	AATGGAG

^a^The Wild type RBSs are shown in bold

^b^“-” indicates base deletion in this location

## Conclusions

In this study, we developed a CRISPR/Cas9n based method for multiplex and iterative genome editing in *B. subtilis*. We performed a detailed characterization of the method and achieved excellent editing efficiency for various types of modifications, especially for large DNA fragment deletions and multiplex point mutations. We also further improved the editing efficiency of CRISPR/Cas9n mediated multiplexing by inhibiting nicks re-ligation. The gene *ligD* was targeted to regulate the nick repair mechanism and thus improve multiplex genome editing. The efficiency for three-point mutations by CRISPR/Cas9n mediated multiplexing system reached 65% in the *ligD* knockout strain. Finally, we applied CRISPR/Cas9n mediated multiplex gene editing to optimize the riboflavin operon for enhanced production of riboflavin in *B. subtilis*. The use of the improved CRISPR/Cas9n system made it possible to modulate multiple genes for metabolic engineering in *B. subtilis*. To our best knowledge, our work offers not only the iterative CRISPR/Cas9n system for *B. subtilis* but also the highest efficiency for simultaneous modulation of multiple genes on the chromosome in *B. subtilis* reported to date.

## Methods

### Strains and culture conditions

All bacterial strains and plasmids used in this work are listed in Table 2. *E. coli*. DH5α was used as the host strain for cloning and plasmid construction. *B. subtilis* 168 was used for the characterization of CRISPR-Cas9/Cas9n mediated genome editing in cells. All other *B. subtilis* strains were derived from the wild-type *B. subtilis*168. Luria–Bertani (LB) medium was used for plasmid construction in *E. coli*. Riboflavin production was tested in YE medium (K_2_HPO_4_ 0.5 g/L, KH_2_PO_4_ 0.5 g/L, (NH_4_)_4_SO_4_ 0.5 g/L, yeast extract 2 g/L, glucose 100 g/L). The transformation of *B. subtilis* was performed using a standard protocol for natural competence [56]. Transformed cells were cultivated on LB agar plates at 37 °C for 24 h. During strain construction, the cultures were grown aerobically at 37 °C in LB medium. When required, antibiotics were added to the media at the following concentrations: 100 µg/mL ampicillin and 10 µg/mL chloramphenicol for *E. coli* selection; 5 µg/mL chloramphenicol and 0.5 µg/mL erythromycin for *B. subtilis* selection. All colonies were collected and stored at − 80 °C as a 15% glycerol stock.

**Table 2:** Strains and plasmids used in this study

Name	Relevant genotype	Source/reference
Strains		
*B. subtilis* 168	Wild-type strain, *trpC2*	BGSC^a^
*B. subtilis* 168Δ*ligD*	*B. subtilis* 168Δ*ligD*	This study
BS89	*B. subtilis*168Δupp ribC*ribO* yhcF*yvrH*ywaA* P43::*ribA*	[55]
BS89Δ*ligD*	BS89Δ*ligD*	This study
CY series strains	Modulation library strains generated with CRISPR/Cas9n technique	This study
*E. coli*. DH5α	F^−^, φ80*lacZ*Δ*M15*, Δ(*lacZYA*-*argF*)*U169*, *deoR*, *recA1*, *endA1*, *hsdR17*(rk^−^, mk^+^), *phoA*, *supE44*, λ^−^, *thi*-*1*, *gyrA96*, *relA1*	Lab stock
Plasmids		
pCas9cur	*E. coli* cloning vector for the express Cas9 protein, Amp^R^	Lab stock
pBSCas9	*B. subtilis*/*E. coli* shuttle vector, Cm^R^ P_*xylA*_-Cas9, P43-gRNA	This study
pBSCas9n	pBSCas9 harboring Cas9 mutation	This study
pDonor	*B. subtilis*/*E. coli* shuttle vector, Cm^R^ Amp^R^, P*manp*-*rep60*gRNA	This study
pHG	Derived from pUC18 for multiple gRNA construction	This study
pUC18	*E. coli* cloning vector, Amp^R^	Lab stock
pEBs-*cop1*	*B. subtilis*/*E. coli* shuttle vector, Em^R^, Amp^R^	Lab stock
pAX01	*B. subtilis* integration vector, P_*xylA*_, Em^R^, Amp^R^	Lab stock
pHP13	*B. subtilis*/*E.coli* shuttle vector,Cm^R^, Em^R^	Lab stock
pBSCas9-*amyE*gRNA	Derived from pBSCas9 for targeting *amyE*	This study
pBSCas9n-*amyE*gRNA	Derived from pBSCas9n for targeting *amyE*	This study
pBSCas9-Ins1 kb*amyE*gRNA	Derived from pBSCas9 for targeting Δ1kb*amyE*	This study
pBSCas9n-Ins1kb*amyE*gRNA	Derived from pBSCas9n for targeting Δ1kb*amyE*	This study
pBSCas9-Ins2kb*amyE*gRNA	Derived from pBSCas9 for targeting Δ2kb*amyE*	This study
pBSCas9n-Ins2kb*amyE*gRNA	Derived from pBSCas9n for targeting Δ2kb*amyE*	This study
pBSCas9-*amyE*/*upp*gRNA	Derived from pBSCas9 for targeting *amyE* and *upp*	This study
pBSCas9n-*amyE*/*upp*gRNA	Derived from pBSCas9n for targeting *amyE* and *upp*	This study
pBSCas9-*amyE*/*upp*/*sigE*gRNA	Derived from pBSCas9 for targeting *amyE*,*upp* and *sigE*	This study
pBSCas9n-*amyE*/*upp*/*sigE*gRNA	Derived from pBSCas9n for targeting *amyE*,*upp* and *sigE*	This study
pBSCas9-LGgRNA	Derived from pBSCas9 for targeting large DNA fragment	This study
pBSCas9n-LGgRNA	Derived from pBSCas9n for targeting large DNA fragment	This study
pDonor-Del1kb	Derived from pDonor for 1 kb deletion	This study
pDonor-Del2kb	Derived from pDonor for 2 kb deletion	This study
pDonor-Del4kb	Derived from pDonor for 4 kb deletion	This study
pDonor-Del6kb	Derived from pDonor for 6 kb deletion	This study
pDonor-Del8kb	Derived from pDonor for 8 kb deletion	This study
pDonor-Ine1kb	Derived from pDonor for 1 kb insertion	This study
pDonor-Ine2kb	Derived from pDonor for 2 kb insertion	This study
pDonor-DelLDNA	Derived from pDonor for large DNA fragment deletion	This study
pDonor-Mu*amyE*	Derived from pDonor for codon replacement in *amyE*	This study
pDonor-Mu*amyE*/*upp*	Derived from pDonor for codon replacement in *amyE* and *upp*	This study
pDonor-Mu*amyE*/*upp*/*sigE*	Derived from pDonor for codon replacement in *amyE*,*upp* and *sigE*	This study
pBSCas9n-gRNArib	Derived from pBSCas9n for targeting *ribB*, *ribA* and *ribH*	This study
pDonor-ribRBSLib	Derived from pDonor for generating a combinatorial library	This study

*Cm* chloramphenicol; *Amp* ampicillin; *Em* erythromycin; *R* resistance

^a^*Bacillus* Genetic Stock Center

### Plasmids construction

To construct the plasmid pBSCas9 (Additional file 1: Figure S1), the promoter P43 was amplified from the genome of *B. subtilis* 168 using the primers P43-F and P43-L, and the pUC18 origin was amplified from plasmid pUC18 using the primers pUC-F and pUC-L. The fragments encoding P43 and the pUC18 replicon were fused using the primers P43-F and pUC-L to generate fragment F1. In this process, the sequence for gRNA-Cas9 binding and a terminator derived from *Streptococcus pyogenes* were synthesized as part of the primers and introduced into the plasmid pBSCas9 backbone. The *cat* gene was amplified from plasmid pHP13 using the primers Cm-F and Cm-L, the *repF* replicon was amplified from pEBs-*cop1* using the primers *repF*-F and *repF*-L, the P*xylA* cassette was amplified from pAX01 using the primers *xylA*-F and *xylA*-L. The fragments encoding the *cat* gene, *repF* replicon and P*xylA* cassette were fused using the primers pUC-F and xylA-L to generate fragment F2. The Cas9 gene was amplified from pCas9cur using the primers Cas9-F and Cas9-L to generate fragment F3. The fragments F1, F2 and F3 were assembled using the CPEC method [57] to generate pBSCas9. To construct the targeting gRNA, a set of primers was used to PCR amplify the pBSCas9 backbone. The 20 bp spacer sequence specific for each target was synthesized as part of the primers. The PCR product was then self-ligated using Golden Gate Assembly [58] to obtain the corresponding plasmid pBSCas9-gRNA. We employed a previously described method to use a single Golden Gate assembly reaction to construct the gRNA plasmid expressing two or three gRNAs simultaneously [21]. The detailed design and procedure are shown in Additional file 2: Figure S2.

To construct the plasmid pDonor (Additional file 1: Figure S1), the backbone of the plasmid pHP13 was amplified iteratively using the primers pairs pDon1-F/L and pDon2-F/L. In this process, the promoter P*manp* and the sequence for gRNA targeting *rep60* replicon were synthesized as part of the primers and introduced, respectively, into pHP13 backbone. The PCR product from each step of PCR was ligated using Golden Gate Assembly, finally resulting in pDonor. To construct the donor DNA, homologous arms flanking the target loci and sequence to be inserted were separately amplified and were then fused by overlap-extension PCR. The fused fragment was introduced into pDonor using the CPEC method.

To construct the Cas9 nickase, the backbone of the plasmid pBSCas9 was amplified using 5′-phosphorylated primers and was then ligated by T4 DNA ligase. In this process, the mutations D10A and H840A were synthesized as part of the primers and introduced, respectively, into the pBSCas9 backbone. All primers and spacers used in this study are listed in Additional file 2: Table S1.

### Iterative genome editing procedure

The competent cells of *B. subtilis* 168 were prepared using previously described method. The two plasmids, respectively, expressing gRNA and donor DNA were introduced into *B. subtilis* successively. The transformants were seeded into LB medium containing chloramphenicol (5 µg/mL), erythromycin (0.5 µg/mL) and 1% xylose and cultivated for 8–10 h. The cultured cells were serially diluted and spread on LB agar plate containing chloramphenicol (5 µg/mL) and erythromycin (0.5 µg/mL). The mutations were confirmed by phenotypic validation, PCR and DNA sequencing. For curing the plasmid pDonor, the positive colony was inoculated in LB medium containing 1% xylose and 1% mannose and cultivated for 12 h (Additional file 2: Figure S3). Then, the colonies that were sensitive to erythromycin were cultured at 50 °C, overnight for curing plasmid pBSCas9 or pBSCas9n [16]. After plasmid curing, the cultures were streaked and colonies were tested for chloramphenicol sensitivity. To save time, we usually inoculated colonies for the next round of editing before testing for chloramphenicol sensitivity because of the high curing efficiency in this step.

### Determining the editing efficiency and number of colony forming units

The *amyE* gene locus was used as the target for gene knockout, gene insertion and single-point mutation. The *amyE* and *upp* genes were targeted for two-point mutations, *amyE*, *upp* and *sigE* were targeted for three simultaneous point mutations. The prophage (-like) region (SubtiList coordinates: 528148–548697) was targeted for large DNA fragment deletion. The editing efficiency was determined by calculating the PCR analytical number of positive colonies. To save time, when two or three-point mutations were implemented, phenotypic analysis (*amyE* and *upp*) was employed to exclude false-positive colonies and then DNA sequencing was performed to confirm that the target genes were modified.

The total number of cells were counted after the xylose-induced cells harboring two plasmids were diluted and spread onto LB agar plates. The CFU were determined by calculating counts per 1 mL based on total liquid volume spread on plates.

### Generation of an RBS-modulation library using CRISPR/Cas9n

The RBS of *ribA* was designed as a typical sequence AAGGAGG in *B. subtilis*, and it was introduced into the genome during the construction of strain BS89. The RBS sequences of *ribB* and *ribH* were determined by sequence analysis of non-coding region upstream of their start codons. They were identified with high homology to GGAGG at about 4 bp upstream of the start codon [59]. To generate a combinatorial library of variably improved riboflavin operon pathway genes, the plasmids pBSCas9n-gRNArib and pDonor-ribRBSLib were constructed. To preserve RBS sequence specificity and basal translation strength in *B. subtilis*, the first one and last two bases were designed as A or G [59], and the others were degenerate. The RBS library was correspondingly designed as semi-degenerate sequences RNNNNRR (N: 25% possibility of each of the A, G, C, and T bases; R: 50% possibility of each of the A and G bases). The RBS library was designed based on donor DNA with random nucleotides (RNNNNRR) in the RBS region. The random nucleotides were provided on primers used to construct the donor DNA plasmid. The upstream and downstream fragments of corresponding genes were obtained by PCR amplification with the genome as template. DNA fragments containing the regulator library were obtained by overlap-extension PCR with upstream and downstream fragments and inserted into pDonor to form pDonor-ribRBSLib using the CPEC method.

### Fermentation conditions

Riboflavin production was carried out in shake-flask cultivation medium. To test the riboflavin biosynthesis activity of the engineered strains, a single colony of each strain was transferred into 5 mL of LB medium and incubated at 41 °C in a rotatory shaker at 240 rpm for 14 h to prepare the inocula. The inocula were added aseptically at 2% (v/v) to a 500 mL shake flask containing 50 mL of shake-flask YE medium. The fermentation was carried out at 41 °C in shake flasks at 240 rpm for 48 h.

### Analytical methods

The growth of *B. subtilis* was monitored by measuring the optical density at 600 nm (OD_600_) using a conventional UV–Vis spectrophotometer. Glucose consumption was quantified using an SBA-40E biosensor (Shandong Province Academy of Sciences, China). For riboflavin measurement, samples were first diluted with 0.05 M NaOH and centrifuged at 1000 *g* for 2 min to remove the cells, after which the supernatant was diluted with acetic acid sodium-acetate buffer (pH 5.0) to the linear range of the spectrophotometer and the absorbance at 444 nm was recorded [60]. The riboflavin concentration was calculated using the standard equation which had been validated, *Y* = (A_444_ − 0.0057) × DF/0.0321 [*R*^2^ = 0.9968; *Y*, the riboflavin concentration of sample (mg/L); A_444_, the value of absorbance at 444 nm; DF, dilution fold; A_444_ was controlled within the range of 0.3-0.8 by dilution].

## Additional files

**Additional file 1: Figure S1.** Maps and sequences of plasmids pBSCas9, pDonor and pHG.

**Additional file 2: Figure S2.** Strategy for the construction of plasmids expressing multiple gRNA sequences. **Figure S3.** CRISPR-Cas9/Cas9n mediated inducible plasmid curing. **Figure S4.** The influence of *ligD* knockout on cell growth. **Figure S5.** The secondary structures in the 5′-UTR regions of the mRNA of riboflavin operon genes in strain BS89. **Figure S6.** The secondary structures in the 5′-UTR regions of the mRNA of riboflavin operon genes in strain CY46. **Figure S7.** The secondary structures in the 5′-UTR regions of the mRNA of riboflavin operon genes in strain CY124. **Figure S8.** The secondary structures in the 5′-UTR regions of the mRNA of riboflavin operon genes in strain CY79. **Figure S9.** The secondary structures in the 5′-UTR regions of the mRNA of riboflavin operon genes in strain CY49. **Table S1.** Primers and gRNA sequences used in this study.

## Data Availability

The materials and data used and/or analyzed during the current study are available from the corresponding author on reasonable request.

## Abbreviations

DSB: double-strand break
SSB: single-strand break
HDR: homology directed repair
CFU: colony forming units
NHEJ: non-homologous end joining
CRISPR: clustered regularly interspaced short palindromic repeats
GRAS: generally regarded as safe
