# Supplementary material for: Development and characterization of a CRISPR/Cas9n-based multiplex genome editing system for Bacillus subtilis
Source: Biotechnol Biofuels. 2019 Sep 27;12:197. doi: 10.1186/s13068-019-1537-1 (PMC6764132; doi:10.1186/s13068-019-1537-1)
Supplement: Supplementary file 1 — Additional file 1: Figure S1. Maps and sequences of plasmids pBSCas9, pDonor and pHG. [file 13068_2019_1537_MOESM1_ESM.docx]

**Additional file 1:**

**Development and Characterization of** **a CRISPR/Cas9n-Based Multiplex Genome Editing System for *Bacillus subtilis***

Dingyu Liu^a#^, Can Huang^a#^, Jiaxin Guo^a^, Peiji Zhang^a^, Tao Chen^a^, Zhiwen Wang^a*^, Xueming Zhao^a^

^a^ Frontier Science Center for Synthetic Biology and Key Laboratory of Systems Bioengineering (Ministry of Education), SynBio Research Platform, Collaborative Innovation Center of Chemical Science and Engineering (Tianjin), School of Chemical Engineering and Technology, Tianjin University, Tianjin, 300072, China.

# These authors contributed equally to this work.

* Corresponding author: Zhiwen Wang.

Tel: +86-22-85356617; Fax: +86-22-85356617.

Address: Department of Biochemical Engineering, School of Chemical Engineering and Technology, Tianjin University, Tianjin 300072, People’s Republic of China.

**Figure S1**

**Map of the plasmid pBSCas9**


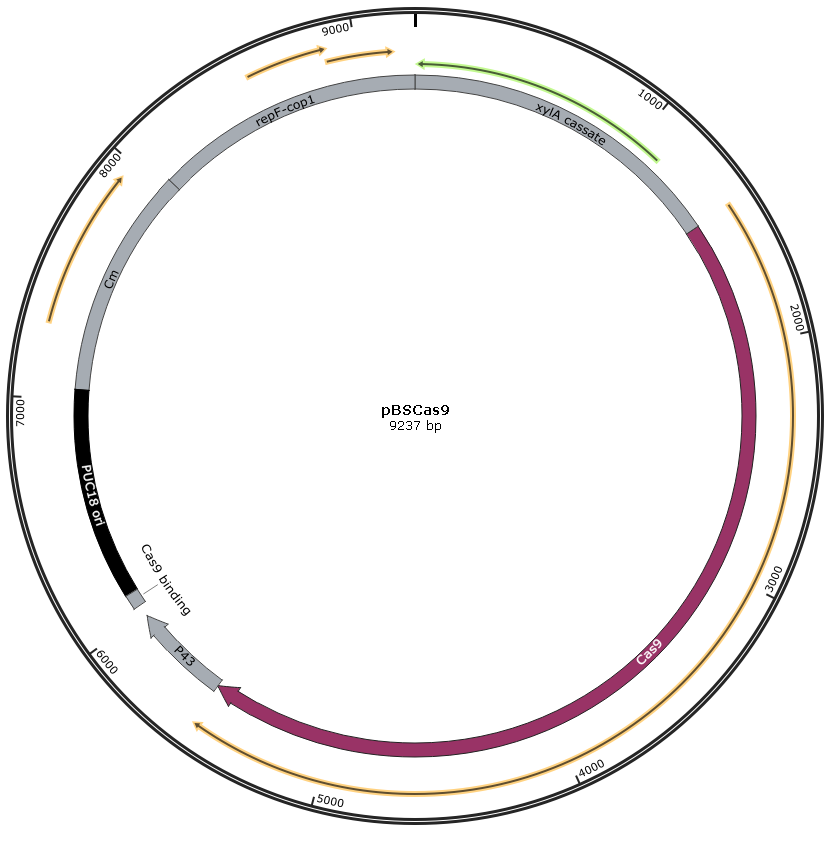


**Sequence of plasmid pBSCas9**

CTAACTTATAGGGGTAACACTTAAAAAAGAATCAATAACGATAGAAACCGCTCCTAAAGCAGGTGCATTTTTTCCTAACGAAGAAGGCAATAGTTCACATTTATTGTCTAAATGAGAATGGACTCTAGAAGAAACTTCGTTTTTAATCGTATTTAAAACAATGGGATGAGATTCAATTATATGATTTCTCAAGATAACAGCTTCTATATCAAATGTATTAAGGATATTGGTTAATCCAATTCCGATATAAAAGCCAAAGTTTTGAAGTGCATTTAACATTTCTACATCATTTTTATTTGCGCGTTCCACAATCTCTTTTCGAGAAATATTCTTTTCTTCTTTAGAGAGCGAAGCCAGTAACGCTTTTTCAGAAGCATATAATTCCCAACAGCCTCGATTTCCACAGCTGCATTTGGGTCCATTAAAATCTATCGTCATATGACCCATTTCCCCAGAAAAACCCTGAACACCTTTATACAATTCGTTGTTAATAACAAGTCCAGTTCCAATTCCGATATTAATACTGATGTAAACGATGTTTTCATAGTTTTTTGTCATACCAAATACTTTTTCACCGTATGCTCCTGCATTAGCTTCATTTTCAACAAAAACCGGAACATTAAACTCACTCTCAATTAAAAACTGCAAATCTTTGATATTCCAATTTAAGTTAGGCATGAAAATAATTTGCTGATGACGATCTACAAGGCCTGGAACACAAATTCCTATTCCGACTAGACCATAAGGGGACTCAGGCATATGGGTTACAAAACCATGAATAAGTGCAAATAAAATCTCTTTTACTTCACTAGCGGAAGAACTAGACAAGTCAGAAGTCTTCTCGAGAATAATATTTCCTTCTAAGTCGGTTAGAATTCCGTTAAGATAGTCGACTCCTATATCAATACCAATCGAGTAGCCTGCATTCTTATTAAAAACAAGCATTACAGGTCTTCTGCCGCCTCTAGATTGCCCTGCCCCAATTTCAAAAATAAAATCTTTTTCAAGCAGTGTATTTACTTGAGAGGAGACAGTAGACTTGTTTAATCCTGTAATCTCAGAGAGAGTTGCCCTGGAGACAGGGGAGTTCTTCAAAATTTCATCTAATATTAATTTTTGATTCATTTTTTTTACTAAAGCTTGATCTGCAATTTGAATAATAACCACTCCTTTGTTTATCCACCGAACTAAGTTGGTGTTTTTTGAAGCTTGAATTAGATATTTAAAAGTATCATATCTAATATTATAACTAAATTTTCTAAAAAAAACATTGAAATAAACATTTATTTTGTATATGATGAGATAAAGTTAGTTTATTGGATAAACAAACTAACTCAATTAAGATAGTTGATGGATAAACTTGTTCACTTAAATCAAAGGGGGAAATGACAAATGGTCCAAACTAGTGATATCTAAAAATCAAAGGGGGAAATGGGATCCATGGATAAGAAATACTCAATAGGCTTAGATATCGGCACAAATAGCGTCGGATGGGCGGTGATCACTGATGAATATAAGGTTCCGTCTAAAAAGTTCAAGGTTCTGGGAAATACAGACCGCCACAGTATCAAAAAAAATCTTATAGGGGCTCTTTTATTTGACAGTGGAGAGACAGCGGAAGCGACTCGTCTCAAACGGACAGCTCGTAGAAGGTATACACGTCGGAAGAATCGTATTTGTTATCTACAGGAGATTTTTTCAAATGAGATGGCGAAAGTAGATGATAGTTTCTTTCATCGACTTGAAGAGTCTTTTTTGGTGGAAGAAGACAAGAAGCATGAACGTCATCCTATTTTTGGAAATATAGTAGATGAAGTTGCTTATCATGAGAAATATCCAACTATCTATCATCTGCGAAAAAAATTGGTAGATTCTACTGATAAAGCGGATTTGCGCTTAATCTATTTGGCCTTAGCGCATATGATTAAGTTTCGTGGTCATTTTTTGATTGAGGGAGATTTAAATCCTGATAATAGTGATGTGGACAAACTATTTATCCAGTTGGTACAAACCTACAATCAATTATTTGAAGAAAACCCTATTAACGCAAGTGGAGTAGATGCTAAAGCGATTCTTTCTGCACGATTGAGTAAATCAAGACGATTAGAAAATCTCATTGCTCAGCTCCCCGGTGAGAAGAAAAATGGCTTATTTGGGAATCTCATTGCTTTGTCATTGGGTTTGACCCCTAATTTTAAATCAAATTTTGATTTGGCAGAAGATGCTAAATTACAGCTTTCAAAAGATACTTACGATGATGATTTAGATAATTTATTGGCGCAAATTGGAGATCAATATGCTGATTTGTTTTTGGCAGCTAAGAATTTATCAGATGCTATTTTACTTTCAGATATCCTAAGAGTAAATACTGAAATAACTAAGGCTCCCCTATCAGCTTCAATGATTAAACGCTACGATGAACATCATCAAGACTTGACTCTTTTAAAAGCTTTAGTTCGACAACAACTTCCAGAAAAGTATAAAGAAATCTTTTTTGATCAATCAAAAAACGGATATGCAGGTTATATTGATGGGGGAGCTAGCCAAGAAGAATTTTATAAATTTATCAAACCAATTTTAGAAAAAATGGATGGTACTGAGGAATTATTGGTGAAACTAAATCGTGAAGATTTGCTGCGCAAGCAACGGACCTTTGACAACGGCTCTATTCCCCATCAAATTCACTTGGGTGAGCTGCATGCTATTTTGAGAAGACAAGAAGACTTTTATCCATTTTTAAAAGACAATCGTGAGAAGATTGAAAAAATCTTGACTTTTCGAATTCCTTATTATGTTGGTCCATTGGCGCGTGGCAATAGTCGTTTTGCATGGATGACTCGGAAGTCTGAAGAAACAATTACCCCATGGAATTTTGAAGAAGTTGTCGATAAAGGTGCTTCAGCTCAATCATTTATTGAACGCATGACAAACTTTGATAAAAATCTTCCAAATGAAAAAGTACTACCAAAACATAGTTTGCTTTATGAGTATTTTACGGTTTATAACGAATTGACAAAGGTCAAATATGTTACTGAAGGAATGCGAAAACCAGCATTTCTTTCAGGTGAACAGAAGAAAGCCATTGTTGATTTACTCTTCAAAACAAATCGAAAAGTAACCGTTAAGCAATTAAAAGAAGATTATTTCAAAAAAATAGAATGTTTTGATAGTGTTGAAATTTCAGGAGTTGAAGATAGATTTAATGCTTCATTAGGTACCTACCATGATTTGCTAAAAATTATTAAAGATAAAGATTTTTTGGATAATGAAGAAAATGAAGATATCTTAGAGGATATTGTTTTAACATTGACCTTATTTGAAGATAGGGAGATGATTGAGGAAAGACTTAAAACATATGCTCACCTCTTTGATGATAAGGTGATGAAACAGCTTAAACGTCGCCGTTATACTGGTTGGGGACGTTTGTCTCGAAAATTGATTAATGGTATTAGGGATAAGCAATCTGGCAAAACAATATTAGATTTTTTGAAATCAGATGGTTTTGCCAATCGCAATTTTATGCAGCTGATCCATGATGATAGTTTGACATTTAAAGAAGACATTCAAAAAGCACAAGTGTCTGGACAAGGCGATAGTTTACATGAACATATTGCAAATTTAGCTGGTAGCCCTGCTATTAAAAAAGGTATTTTACAGACTGTAAAAGTTGTTGATGAATTGGTCAAAGTAATGGGGCGGCATAAGCCAGAAAATATCGTTATTGAAATGGCACGTGAAAATCAGACAACTCAAAAGGGCCAGAAAAATTCGCGAGAGCGTATGAAACGAATCGAAGAAGGTATCAAAGAATTAGGAAGTCAGATTCTTAAAGAGCATCCTGTTGAAAATACTCAATTGCAAAATGAAAAGCTCTATCTCTATTATCTCCAAAATGGAAGAGACATGTATGTGGACCAAGAATTAGATATTAATCGTTTAAGTGATTATGATGTCGATCACATTGTTCCACAAAGTTTCCTTAAAGACGATTCAATAGACAATAAGGTCTTAACGCGTTCTGATAAAAATCGTGGTAAATCGGATAACGTTCCAAGTGAAGAAGTAGTCAAAAAGATGAAAAACTATTGGAGACAACTTCTAAACGCCAAGTTAATCACTCAACGTAAGTTTGATAATTTAACGAAAGCTGAACGTGGAGGTTTGAGTGAACTTGATAAAGCTGGTTTTATCAAACGCCAATTGGTTGAAACTCGCCAAATCACTAAGCATGTGGCACAAATTTTGGATAGTCGCATGAATACTAAATACGATGAAAATGATAAACTTATTCGAGAGGTTAAAGTGATTACCTTAAAATCTAAATTAGTTTCTGACTTCCGAAAAGATTTCCAATTCTATAAAGTACGTGAGATTAACAATTACCATCATGCCCATGATGCGTATCTAAATGCCGTCGTTGGAACTGCTTTGATTAAGAAATATCCAAAACTTGAATCGGAGTTTGTCTATGGTGATTATAAAGTTTATGATGTTCGTAAAATGATTGCTAAGTCTGAGCAAGAAATAGGCAAAGCAACCGCAAAATATTTCTTTTACTCTAATATCATGAACTTCTTCAAAACAGAAATTACACTTGCAAATGGAGAGATTCGCAAACGCCCTCTAATCGAAACTAATGGGGAAACTGGAGAAATTGTCTGGGATAAAGGGCGAGATTTTGCCACAGTGCGCAAAGTATTGTCCATGCCCCAAGTCAATATTGTCAAGAAAACAGAAGTACAGACAGGCGGATTCTCCAAGGAGTCAATTTTACCAAAAAGAAATTCGGACAAGCTTATTGCTCGTAAAAAAGACTGGGATCCAAAAAAATATGGTGGTTTTGATAGTCCAACGGTAGCTTATTCAGTCCTAGTGGTTGCTAAGGTGGAAAAAGGGAAATCGAAGAAGTTAAAATCCGTTAAAGAGTTACTAGGGATCACAATTATGGAAAGAAGTTCCTTTGAAAAAAATCCGATTGACTTTTTAGAAGCTAAAGGATATAAGGAAGTTAAAAAAGACTTAATCATTAAACTACCTAAATATAGTCTTTTTGAGTTAGAAAACGGTCGTAAACGGATGCTGGCTAGTGCCGGAGAATTACAAAAAGGAAATGAGCTGGCTCTGCCAAGCAAATATGTGAATTTTTTATATTTAGCTAGTCATTATGAAAAGTTGAAGGGTAGTCCAGAAGATAACGAACAAAAACAATTGTTTGTGGAGCAGCATAAGCATTATTTAGATGAGATTATTGAGCAAATCAGTGAATTTTCTAAGCGTGTTATTTTAGCAGATGCCAATTTAGATAAAGTTCTTAGTGCATATAACAAACATAGAGACAAACCAATACGTGAACAAGCAGAAAATATTATTCATTTATTTACGTTGACGAATCTTGGAGCTCCCGCTGCTTTTAAATATTTTGATACAACAATTGATCGTAAACGATATACGTCTACAAAAGAAGTTTTAGATGCCACTCTTATCCATCAATCCATCACTGGTCTTTATGAAACACGCATTGATTTGAGTCAGCTAGGAGGTGACTGACGTGCATGCAGGCCGGGGCATATGGGAAACAGCGCGGACGCAGCGGAATTTCCAATTTCATGCCGCAGCCGCCTGCGCTGTTCTCATTTGCGGCTTCCTTGTAGAGCTCAGCATTATTGAGTGGATGATTATATTCCTTTTGATAGGTGGTATGTTTTCGCTTGAACTTTTAAATACAGCCATTGAACATACGGTTGATTTAATAACTGACAAACATCACCCTCTTGCTAAAGCGGCCAAGGACGCTGCCGCCGGGGCTGTTTGCGTTTTTGCCGTGATTTCGTGTATCATTGGTTTACTTATTTTTTTGCCAAAGCTGTAATGGCTGAAAATTCTTACATTTATTTTACATTTTTAGAAATGGGCGTGAAAAAAAGCGCGCGATTATGTAAAATATAAAGTGATAGCGGTACCATTATAGGTAAGAGAGGAATGTACACgtttcggtgatgacctttccagtcgggaaacctgtcGTAGGTCGTTCGCTCCAAGCGTTTTAGAGCTAGAAATAGCAAGTTAAAATAAGGCTAGTCCGTTATCAACTTGAAAAAGTGGCACCCTTCCTCGCTCACTGACTCGCTGCGCTCGGTCGTTCGGCTGCGGCGAGCGGTATCAGCTCACTCAAAGGCGGTAATACGGTTATCCACAGAATCAGGGGATAACGCAGGAAAGAACATGTGAGCAAAAGGCCAGCAAAAGGCCAGGAACCGTAAAAAGGCCGCGTTGCTGGCGTTTTTCCATAGGCTCCGCCCCCCTGACGAGCATCACAAAAATCGACGCTCAAGTCAGAGGTGGCGAAACCCGACAGGACTATAAAGATACCAGGCGTTTCCCCCTGGAAGCTCCCTCGTGCGCTCTCCTGTTCCGACCCTGCCGCTTACCGGATACCTGTCCGCCTTTCTCCCTTCGGGAAGCGTGGCGCTTTCTCATAGCTCACGCTGTAGGTATCTCAGTTCGGTGTAGGTCGTTCGCTCCAAGCTGGGCTGTGTGCACGAACCCCCCGTTCAGCCCGACCGCTGCGCCTTATCCGGTAACTATCGTCTTGAGTCCAACCCGGTAAGACACGACTTATCGCCACTGGCAGCAGCCACTGGTAACAGGATTAGCAGAGCGAGGTATGTAGGCGGTGCTACAGAGTTCTTGAAGTGGTGGCCTAACTACGGCTACACTAGAAGGACAGTATTTGGTATCTGCGCTCTGCTGAAGCCAGTTACCTTCGGAAAAAGAGTTGGTAGCTCTTGATCCGGCAAACAAACCACCGCTGGTAGCGGTGGTTTTTTTGTTTGCAAGCAGCAGATTACGCGCAGAAAAAAAGGATCTCAAGAAGATCCTTTGATCTTTTCTACGGGGTCTGACGCTCAGTGGAACGAAAACTCACGTTAAGGGATTTTGGTCATGAGATTATCAAAAAGGATCTTCACCTAGATCCTTTTAAATTAAAAATGAAGTTTTAAATCAATCTAAAGTATATATGAGTAAACTTGGTCTGACAGAAAGGATTTTTCGCTACGCTCAAATCCTTTAAAAAAACACAAAAGACCACATTTTTTAATGTGGTCTTTATTCTTCAACTAAAGCACCCATTAGTTCAACAAACGAAAATTGGATAAAGTGGGATATTTTTAAAATATATATTTATGTTACAGTAATATTGACTTTTAAAAAAGGATTGATTCTAATGAAGAAAGCAGACAAGTAAGCCTCCTAAATTCACTTTAGATAAAAATTTAGGAGGCATATCAAATGAACTTTAATAAAATTGATTTAGACAATTGGAAGAGAAAAGAGATATTTAATCATTATTTGAACCAACAAACGACTTTTAGTATAACCACAGAAATTGATATTAGTGTTTTATACCGAAACATAAAACAAGAAGGATATAAATTTTACCCTGCATTTATTTTCTTAGTGACAAGGGTGATAAACTCAAATACAGCTTTTAGAACTGGTTACAATAGCGACGGAGAGTTAGGTTATTGGGATAAGTTAGAGCCACTTTATACAATTTTTGATGGTGTATCTAAAACATTCTCTGGTATTTGGACTCCTGTAAAGAATGACTTCAAAGAGTTTTATGATTTATACCTTTCTGATGTAGAGAAATATAATGGTTCGGGGAAATTGTTTCCCAAAACACCTATACCTGAAAATGCTTTTTCTCTTTCTATTATTCCATGGACTTCATTTACTGGGTTTAACTTAAATATCAATAATAATAGTAATTACCTTCTACCCATTATTACAGCAGGAAAATTCATTAATAAAGGTAATTCAATATATTTACCGCTATCTTTACAGGTACATCATTCTGTTTGTGATGGTTATCATGCAGGATTGTTTATGAACTCTATTCAGGAATTGTCAGATAGGCCTAATGACTGGCTTTTATAATATGAGATAATGCCGACTGTACTTTTTACAGTCGGTTTTCTAATGTCACTAACCTGCCCCGTTAGTTGAAGAAGGTTTTTATATTACAGCTCCAGATCCATATCCTTTAATGGACGCAGCGCATCACACGCAAAAAGGAAATTGGAATAAATGCGAAATTTGAGATGTTAATTAAAGACCTTTTTGAGGTCTTTTTTTCTTAGATTTTTGGGGTTATTTAGGGGAGAAAACATAGGGGGGTACTACGACCTCCCCCCTAGGTGTCCATTGTCCATTGTCCAAACAAATAAATAAATATTGGGTTTTTAATGTTAAAAGGTTGTTTTTTATGTTAAAGTGAAAAAAACAGATGTTGGGAGGTACAGTGATGGTTGTAGATAGAAAAGAAGAGAAAAAAGTTGCTGTTACTTTAAGACTTACAACAGAAGAAAATGAGATATTAAATAGAATCAAAGAAAAATATAATATTAGCAAATCAGATGCAACCGGTATTCTAATAAAAAAATATGCAAAGGAGGAATACGGTGCATTTTAAACAAAAAAAGATAGACAGCACTGGCATGCTGCCTATCTATGACTAAATTTTGTTAAGTGTATTAGCACCGTTATTATATGATGAGCGAAAATGTAATAAAAGAAACTGAAAACAAGAAAAATTCAAGAGGACGTAATTGGACATTTGTTTTATATCCAGAATCAGCAAAAGCCGAGTGGTTAGAGTATTTAAAAGAGTTACACATTCAATTTGTAGTGTCTCCATTACATGATAGGGATACTGATACAGAAGGTAGGATGAAAAAAGAGCATTATCATATTCTAGTGATGTATGAGGGTAATAAATCTTATGAACAGATAAAAATAATTAACAGAAGAATTGAATGCGACTATTCCGCAGATTGCAGGAAGTGTGAAAGGTCTTGTGAGATATATGCTTCACATGGACGATCCTAATAAATTTAAATATCAAAAAGAAGATATGATAGTTTATGGCGGTGTAGATGTTGATGAATTATTAAAGAAAACAACAACAGATAGATATAAATTAATTAAAGAAATGATTGAGTTTATTGATGAACAAGGAATCGTAGAATTTAAGAGTTTAATGGATTATGCAATGAAGTTTAAATTTGATGATTGGTTCCCGCTTTTATGTGATAACTCGGCGTATGTTATTCAAGAATATATAAAATCAAATCGGTATAAATCTGACCGATAGATTTTGAATTTAGGTGTCACAAGACACTCTTTTTTCGCACCAGCGAAAACTGGTTTAAGCCGACTGCGCAAAAGACA

**Map of the plasmid pDonor**


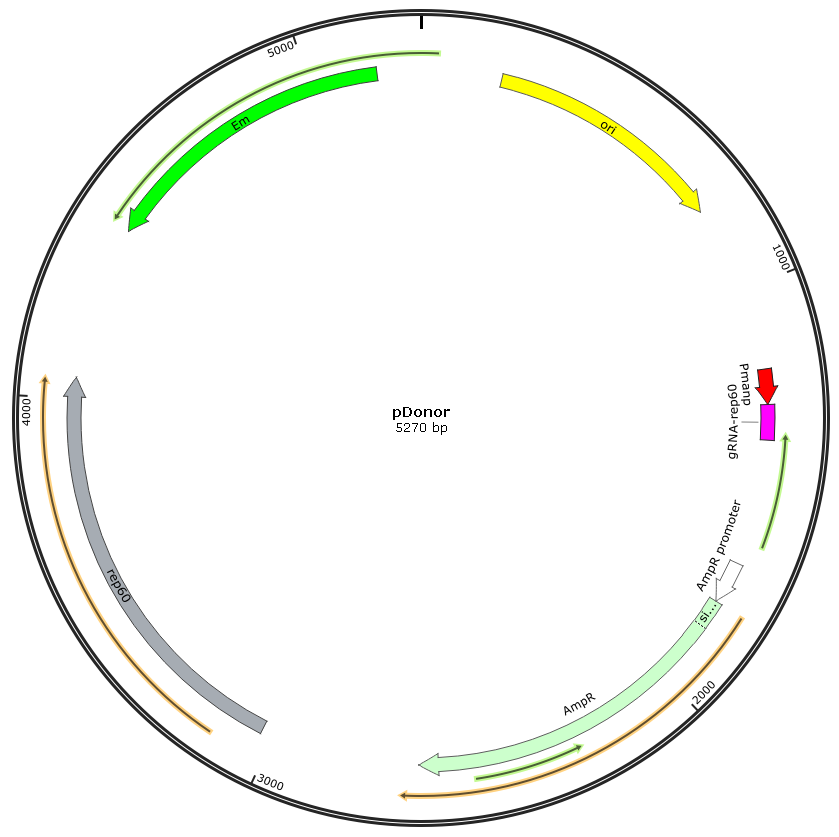


**Sequence of plasmid pDonor**

ggttgataatgaactgtgctgattacaaaaatactaaaaatgcccatattttttcctccttataaaattagtataattatagcacgaaaaggatctaggtgaagatcctttttgataatctcatgaccaaaatcccttaacgtgagttttcgttccactgagcgtcagaccccgtagaaaagatcaaaggatcttcttgagatcctttttttctgcgcgtaatctgctgcttgcaaacaaaaaaaccaccgctaccagcggtggtttgtttgccggatcaagagctaccaactctttttccgaaggtaactggcttcagcagagcgcagataccaaatactgttcttctagtgtagccgtagttaggccaccacttcaagaactctgtagcaccgcctacatacctcgctctgctaatcctgttaccagtggctgctgccagtggcgataagtcgtgtcttaccgggttggactcaagacgatagttaccggataaggcgcagcggtcgggctgaacggggggttcgtgcacacagcccagcttggagcgaacgacctacaccgaactgagatacctacagcgtgagctatgagaaagcgccacgcttcccgaagggagaaaggcggacaggtatccggtaagcggcagggtcggaacaggagagcgcacgagggagcttccagggggaaacgcctggtatctttatagtcctgtcgggtttcgccacctctgacttgagcgtcgatttttgtgatgctcgtcaggggggcggagcctatggaaaaacgccagcaacgcggcctttttacggttcctggccttttgctggccttttgctcacatgttctttcctgcgttatcccctgattctgtggataaccgtattaccgcctttgagtgagctgataccgctcgccgcagccgaacgaccgagcgcagcgagtcagtgagcgaggaagcggaagagcgcccaatacgcaaaccgcctctccccgcgcgttggccgattcattaatgcagctggcacgacaggtttcccgactggaaagcgggcagtgagcgcaacgcaattaatgtgagttagctcactcattaggcaccccaggctttacactttatgcttccggctcgtatgttgtgtggaattgtgagcggataacaatttcacacaggaaacagctatgaccatgattacgccAGGGAAAAATGCCTTTATTACCGGAACCTATGGTAAAAAAAGCGATTTTAATGAGCTGATTTCGGTATACAGTTGAGACAAGATCTgctgacgattcgaaatgtaagttttagagctagaaatagcaagttaaaataaggctagtccgttatcaacttgaaaaagtggcaccaagcttggctgcaggtcgacggatccccgggaattcactggccgtcgttttacaacgtcgtgactgggaaaaccctggcgttacccaacttaatcgccttgcagcacatccccctttcgccagctggcgtaatagcgaagaggcccgcaccgatcgcccttcccaacagttgcgcagcctgaatggcgaatggcgactaacggggcaggttagtgacattaGCCTCGTGATACGCCTATTTTTATAGGTTAATGTCATGATAATAATGGTTTCTTAGACGTCAGGTGGCACTTTTCGGGGAAATGTGCGCGGAACCCCTATTTGTTTATTTTTCTAAATACATTCAAATATGTATCCGCTCATGAGACAATAACCCTGATAAATGCTTCAATAATATTGAAAAAGGAAGAGTATGAGTATTCAACATTTCCGTGTCGCCCTTATTCCCTTTTTTGCGGCATTTTGCCTTCCTGTTTTTGCTCACCCAGAAACGCTGGTGAAAGTAAAAGATGCTGAAGATCAGTTGGGTGCACGAGTGGGTTACATCGAACTGGATCTCAACAGCGGTAAGATCCTTGAGAGTTTTCGCCCCGAAGAACGTTTTCCAATGATGAGCACTTTTAAAGTTCTGCTATGTGGCGCGGTATTATCCCGTATTGACGCCGGGCAAGAGCAACTCGGTCGCCGCATACACTATTCTCAGAATGACTTGGTTGAGTACTCACCAGTCACAGAAAAGCATCTTACGGATGGCATGACAGTAAGAGAATTATGCAGTGCTGCCATAACCATGAGTGATAACACTGCGGCCAACTTACTTCTGACAACGATCGGAGGACCGAAGGAGCTAACCGCTTTTTTGCACAACATGGGGGATCATGTAACTCGCCTTGATCGTTGGGAACCGGAGCTGAATGAAGCCATACCAAACGACGAGCGTGACACCACGATGCCTGTAGCAATGGCAACAACGTTGCGCAAACTATTAACTGGCGAACTACTTACTCTAGCTTCCCGGCAACAATTAATAGACTGGATGGAGGCGGATAAAGTTGCAGGACCACTTCTGCGCTCGGCCCTTCCGGCTGGCTGGTTTATTGCTGATAAATCTGGAGCCGGTGAGCGTGGGTCACGCGGTATCATTGCAGCACTGGGGCCAGATGGTAAGCCCTCCCGTATCGTAGTTATCTACACGACGGGGAGTCAGGCAACTATGGATGAACGAAATAGACAGATCGCTGAGATAGGTGCCTCACTGATTAAGCATTGGaggaggcttacttgtctgctttcttcattagaatcaatccttttttaaaagtcaatattactgtaacataaatatatattttaaaaatatcccactttatccaattttcgtttgttgaactaatgggtgctttagttgaagaataaaagaccacattaaaaaatgtggtcttttgtgtttttttaaaggatttgagcgtagcgaaaaatccttttctttcttatcttgatactatatagaaacaacatcatttttcaaaattaggtcaaagccttgtgtatcaagggtttgatggttctttgacaggtaaaaactccttctgctattattaaggtgtcgaatcaaaataatagaatgctagagaactagctcagaaggagtttttttgttgatttattcatctgaaaatgattatagcatcctcgaagataaaaccgcaacaggtaaaaagcgggattggaaggggaaaaagagacggacgaacctcatggcggagcattacgaagcgttagagagtaagattggggcaccttactatggcaaaaaggctgaaaaactaattagttgtgcagagtatctttcgtttaagagggatccggagacgggcaagttaaaactgtatcaagcccatttttgtaaagtgaggttatgtccgatgtgtgcgtggcgcaggtcgttaaaaattgcttatcacaataagttgatcgtagaggaagccaatagacagtacggctgcggatggatttttctcacgctgacgattcgaaatgtaaagggagaacggctgaagccacaaatttctgcgatgatggaaggctttaggaaactgttccagtacaaaaaagtaaaaacttcggttcttggatttttcagagctttagagattaccaaaaatcatgaagaagatacatatcatcctcattttcatgtgttgataccagtaaggaaaaattattttgggaaaaactatattaagcaggcggagtggacgagcctttggaaaaaggcgatgaaattggattacactccaattgtcgatattcgtcgagtgaaaggtaaagctaagattgacgctgaacagattgaaaacgatgtgcggaacgcaatgatggagcaaaaagctgttctcgaaatctctaaatatccggttaaggatacggatgttgtgcgcggtaataaggtgactgaagacaatctgaacacggtgctttacttggatgatgcgttggcagctcgaaggttaattggatacggtggcattttgaaggagatacataaagagctgaatcttggtgatgcggaggacggcgatctggtcaagattgaggaagaagatgacgaggttgcaaatggtgcatttgaggttatggcttattggcatcctggcattaaaaattacataatcaaataaaaaaagcagacctttagaaggcctgcttttttaactaacccatttgtattgtgttgaaatatgttttgtatggtgcactctcagtacaatctgctctgatgccgcatagttaagccagccccgacacccgccaacacccgctgacgcgccctgacgggcttgtctgctcccggcatccgcttacagacaagctgtgaccgtctccgggagctgcatgtgtcagaggttttcaccgtcatcaccgaaacgcgcgagacgaaagggcctcgtgatacgcctatttttataggttaatgtcatgataataatggtttcttagcgattcacaaaaaataggcacacgaaaaacaagttaagggatgcagtttatgcatcccttacttattaaataatttatagctattgaaaagagataagaattgttcaaagctaatattgtttaaatcgtcaattcctgcatgttttaaggaattgttaaattgattttttgtaaatattttcttgtattctttgttaacccatttcataacgaaataattatacttttgtttatctttgtgtgatattcttgatttttttctacttaatctgataagtgagctattcactttaggtttaggatgaaaatattctcttggaaccatacttaatatagaaatatcaacttctgccattaaaagtaatgccaatgagcgttttgtatttaataatcttttagcaaacccgtattccacgattaaataaatctcattagctatactatcaaaaacaattttgcgtattatatccgtacttatgttataaggtatattaccatatattttataggattggtttttaggaaatttaaactgcaatatatccttgtttaaaacttggaaattatcgtgatcaacaagtttattttctgtagttttgcataatttatggtctatttcaatggcagttacgaaattacacctctttactaattcaagggtaaaatggccttttcctgagccgatttcaaagatattatcatgttcatttaatcttatatttgtcattattttatctatattatgttttgaagtaataaagttttgactgtgttttatatttttctcgttcattataaccctctttaatttggttatatgaattttgcttattaacgattcattataaccacttattttttgtttggttgataatgaactgtgctgattacaaaaatac

**Map of the plasmid pHG**


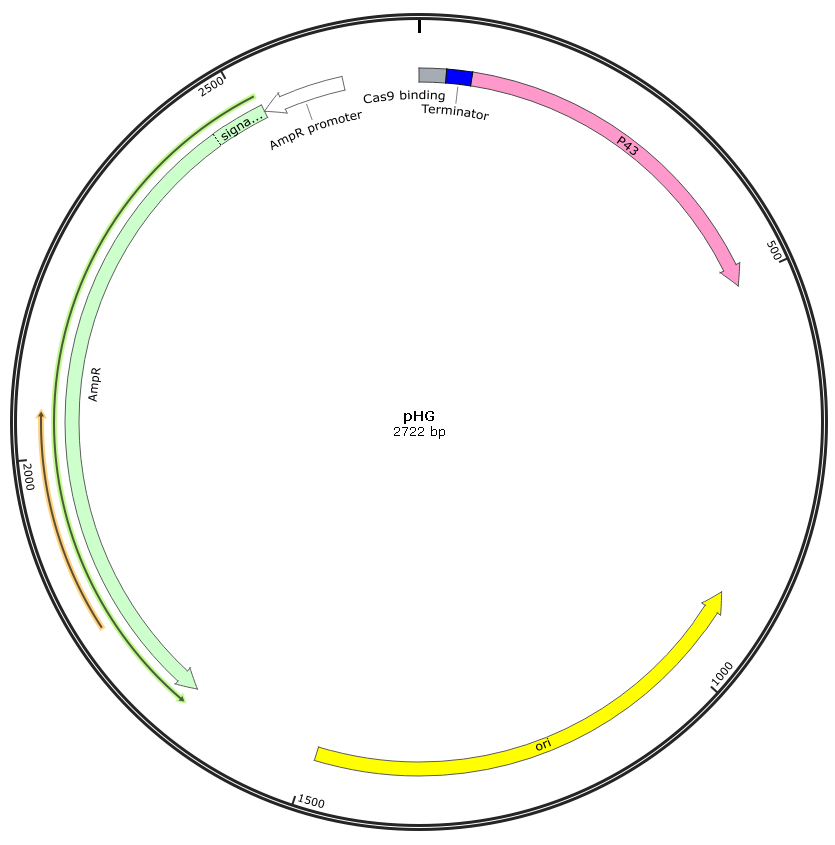


**Sequence of plasmid pHG**

GTTTTAGAGCTAGAAATAGCAAGTTAAAATAAGGCTAGTCCGTTATCAACTTGAAAAAGTGGCACCCGTGCATGCAGGCCGGGGCATATGGGAAACAGCGCGGACGCAGCGGAATTTCCAATTTCATGCCGCAGCCGCCTGCGCTGTTCTCATTTGCGGCTTCCTTGTAGAGCTCAGCATTATTGAGTGGATGATTATATTCCTTTTGATAGGTGGTATGTTTTCGCTTGAACTTTTAAATACAGCCATTGAACATACGGTTGATTTAATAACTGACAAACATCACCCTCTTGCTAAAGCGGCCAAGGACGCTGCCGCCGGGGCTGTTTGCGTTTTTGCCGTGATTTCGTGTATCATTGGTTTACTTATTTTTTTGCCAAAGCTGTAATGGCTGAAAATTCTTACATTTATTTTACATTTTTAGAAATGGGCGTGAAAAAAAGCGCGCGATTATGTAAAATATAAAGTGATAGCGGTACCATTATAGGTAAGAGAGGAATGTACACGCTGTTTCCTGTGTGAAATTGTTATCCGCTCACAATTCCACACAACATACGAGCCGGAAGCATAAAGTGTAAAGCCTGGGGTGCCTAATGAGTGAGCTAACTCACATTAATTGCGTTGCGCTCACTGCCCGCTTTCCAGTCGGGAAACCTGTCGTGCCAGCTGCATTAATGAATCGGCCAACGCGCGGGGAGAGGCGGTTTGCGTATTGGGCGCTCTTCCGCTTCCTCGCTCACTGACTCGCTGCGCTCGGTCGTTCGGCTGCGGCGAGCGGTATCAGCTCACTCAAAGGCGGTAATACGGTTATCCACAGAATCAGGGGATAACGCAGGAAAGAACATGTGAGCAAAAGGCCAGCAAAAGGCCAGGAACCGTAAAAAGGCCGCGTTGCTGGCGTTTTTCCATAGGCTCCGCCCCCCTGACGAGCATCACAAAAATCGACGCTCAAGTCAGAGGTGGCGAAACCCGACAGGACTATAAAGATACCAGGCGTTTCCCCCTGGAAGCTCCCTCGTGCGCTCTCCTGTTCCGACCCTGCCGCTTACCGGATACCTGTCCGCCTTTCTCCCTTCGGGAAGCGTGGCGCTTTCTCATAGCTCACGCTGTAGGTATCTCAGTTCGGTGTAGGTCGTTCGCTCCAAGCTGGGCTGTGTGCACGAACCCCCCGTTCAGCCCGACCGCTGCGCCTTATCCGGTAACTATCGTCTTGAGTCCAACCCGGTAAGACACGACTTATCGCCACTGGCAGCAGCCACTGGTAACAGGATTAGCAGAGCGAGGTATGTAGGCGGTGCTACAGAGTTCTTGAAGTGGTGGCCTAACTACGGCTACACTAGAAGGACAGTATTTGGTATCTGCGCTCTGCTGAAGCCAGTTACCTTCGGAAAAAGAGTTGGTAGCTCTTGATCCGGCAAACAAACCACCGCTGGTAGCGGTGGTTTTTTTGTTTGCAAGCAGCAGATTACGCGCAGAAAAAAAGGATCTCAAGAAGATCCTTTGATCTTTTCTACGGGGTCTGACGCTCAGTGGAACGAAAACTCACGTTAAGGGATTTTGGTCATGAGATTATCAAAAAGGATCTTCACCTAGATCCTTTTAAATTAAAAATGAAGTTTTAAATCAATCTAAAGTATATATGAGTAAACTTGGTCTGACAGTTACCAATGCTTAATCAGTGAGGCACCTATCTCAGCGATCTGTCTATTTCGTTCATCCATAGTTGCCTGACTCCCCGTCGTGTAGATAACTACGATACGGGAGGGCTTACCATCTGGCCCCAGTGCTGCAATGATACCGCGAGACCCACGCTCACCGGCTCCAGATTTATCAGCAATAAACCAGCCAGCCGGAAGGGCCGAGCGCAGAAGTGGTCCTGCAACTTTATCCGCCTCCATCCAGTCTATTAATTGTTGCCGGGAAGCTAGAGTAAGTAGTTCGCCAGTTAATAGTTTGCGCAACGTTGTTGCCATTGCTACAGGCATCGTGGTGTCACGCTCGTCGTTTGGTATGGCTTCATTCAGCTCCGGTTCCCAACGATCAAGGCGAGTTACATGATCCCCCATGTTGTGCAAAAAAGCGGTTAGCTCCTTCGGTCCTCCGATCGTTGTCAGAAGTAAGTTGGCCGCAGTGTTATCACTCATGGTTATGGCAGCACTGCATAATTCTCTTACTGTCATGCCATCCGTAAGATGCTTTTCTGTGACTGGTGAGTACTCAACCAAGTCATTCTGAGAATAGTGTATGCGGCGACCGAGTTGCTCTTGCCCGGCGTCAATACGGGATAATACCGCGCCACATAGCAGAACTTTAAAAGTGCTCATCATTGGAAAACGTTCTTCGGGGCGAAAACTCTCAAGGATCTTACCGCTGTTGAGATCCAGTTCGATGTAACCCACTCGTGCACCCAACTGATCTTCAGCATCTTTTACTTTCACCAGCGTTTCTGGGTGAGCAAAAACAGGAAGGCAAAATGCCGCAAAAAAGGGAATAAGGGCGACACGGAAATGTTGAATACTCATACTCTTCCTTTTTCAATATTATTGAAGCATTTATCAGGGTTATTGTCTCATGAGCGGATACATATTTGAATGTATTTAGAAAAATAAACAAATAGGGGTTCCGCGCACATTTCCCCGAAAAGTGCCACCTGACGTCTAAGAAACCATTATTATCATGACATTAACCTATAAAAATAGGCGTATCACGAGGCCCTTTCGTC
