# Supplementary material for: Development and characterization of a CRISPR/Cas9n-based multiplex genome editing system for Bacillus subtilis
Source: Biotechnol Biofuels. 2019 Sep 27;12:197. doi: 10.1186/s13068-019-1537-1 (PMC6764132; doi:10.1186/s13068-019-1537-1)
Supplement: Supplementary file 2 — Additional file 2: Figure S2. Strategy for the construction of plasmids expressing multiple gRNA sequences. Figure S3. CRISPR-Cas9/Cas9n mediated inducible plasmid curing. Figure S4. The influence of ligD knockout on cell growth. Figure S5. The secondary structures in the 5′-UTR regions of the mRNA of riboflavin operon genes in strain BS89. Figure S6. The secondary structures in the 5′-UTR regions of the mRNA of riboflavin operon genes in strain CY46. Figure S7. The secondary structures in the 5′-UTR regions of the mRNA of riboflavin operon genes in strain CY124. Figure S8. The secondary structures in the 5′-UTR regions of the mRNA of riboflavin operon genes in strain CY79. Figure S9. The secondary structures in the 5′-UTR regions of the mRNA of riboflavin operon genes in strain CY49. Table S1. Primers and gRNA sequences used in this study. [file 13068_2019_1537_MOESM2_ESM.docx]

**Additional file 2:**

**Development and Characterization of** **a CRISPR/Cas9n-Based Multiplex Genome Editing System for *Bacillus subtilis***

Dingyu Liu^a#^, Can Huang^a#^, Jiaxin Guo^a^, Peiji Zhang^a^, Tao Chen^a^, Zhiwen Wang^a*^, Xueming Zhao^a^

^a^ Frontier Science Center for Synthetic Biology and Key Laboratory of Systems Bioengineering (Ministry of Education), SynBio Research Platform, Collaborative Innovation Center of Chemical Science and Engineering (Tianjin), School of Chemical Engineering and Technology, Tianjin University, Tianjin, 300072, China.

# These authors contributed equally to this work.

* Corresponding author: Zhiwen Wang.

Tel: +86-22-85356617; Fax: +86-22-85356617.

Address: Department of Biochemical Engineering, School of Chemical Engineering and Technology, Tianjin University, Tianjin 300072, People’s Republic of China.

**Figure S2**


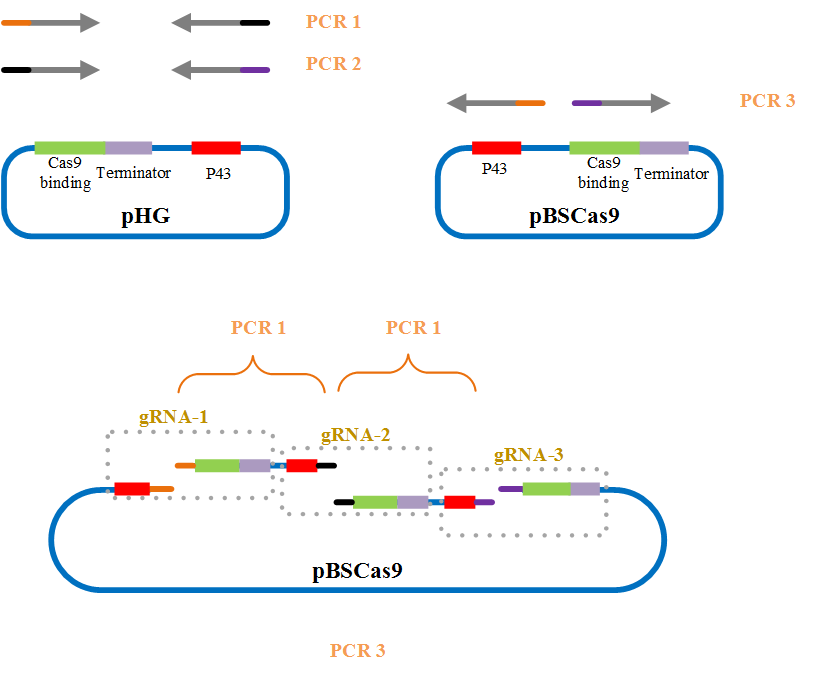


**Figure S2.** Strategy for the construction of plasmids expressing multiple gRNA sequences. PCR 1 and PCR 2 were performed using pHG plasmid (Additional file 1: Figure S1)as template while PCR 3 using pBSCas9 as template. The spacer sequences were synthesized in primers. These three PCR products were assembled using Golden Gate Assembly method, resulting in plasmid expressing three gRNA sequences. It is worth noting that primers used for constructing plasmids expressing a single piece of gRNA can be directly used without any modification for constructing plasmid expressing multiple gRNA sequences.

**Figure S3**


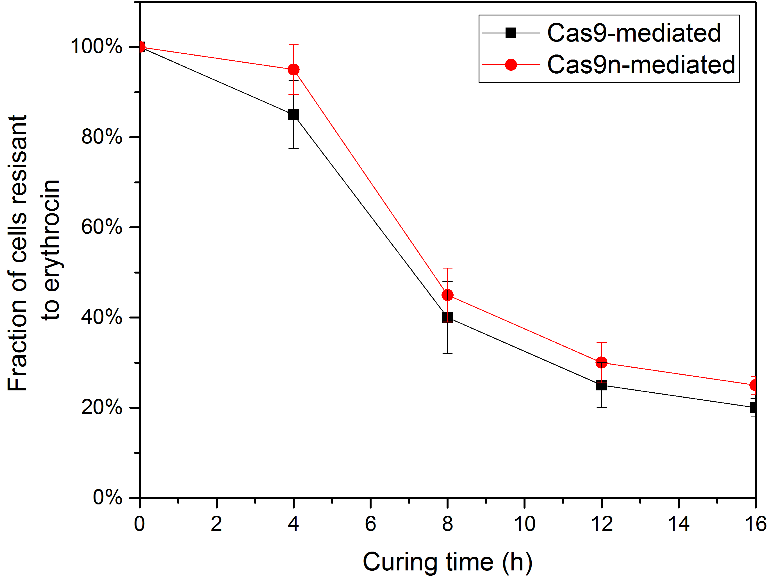


**Figure S3.** CRISPR-Cas9/Cas9n mediated inducible plasmid curing. A single colony was inoculated in LB medium containing 1% xylose and 1% mannose and chloramphenicol. Cells were cultivated at 37 °C for different period before plating on chloramphenicol plates. The resulting colonies were spotted on erythromycin plates to test the loss of plasmid pDonor.

**Figure S4**

**
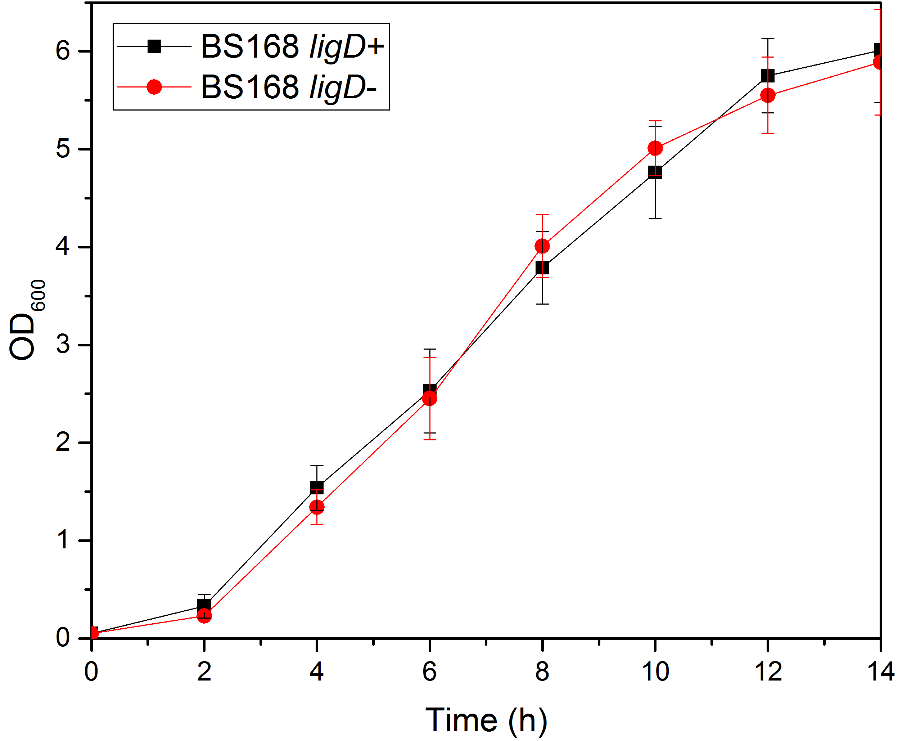
**

**Figure S4.** The influence of *ligD* knockout on cell growth. The cell growth curves of *B. subtilis* 168 and *B. subtilis* 168Δ*ligD* were determined in M9 medium with 20 g/L glucose.

**Figure S5**

**
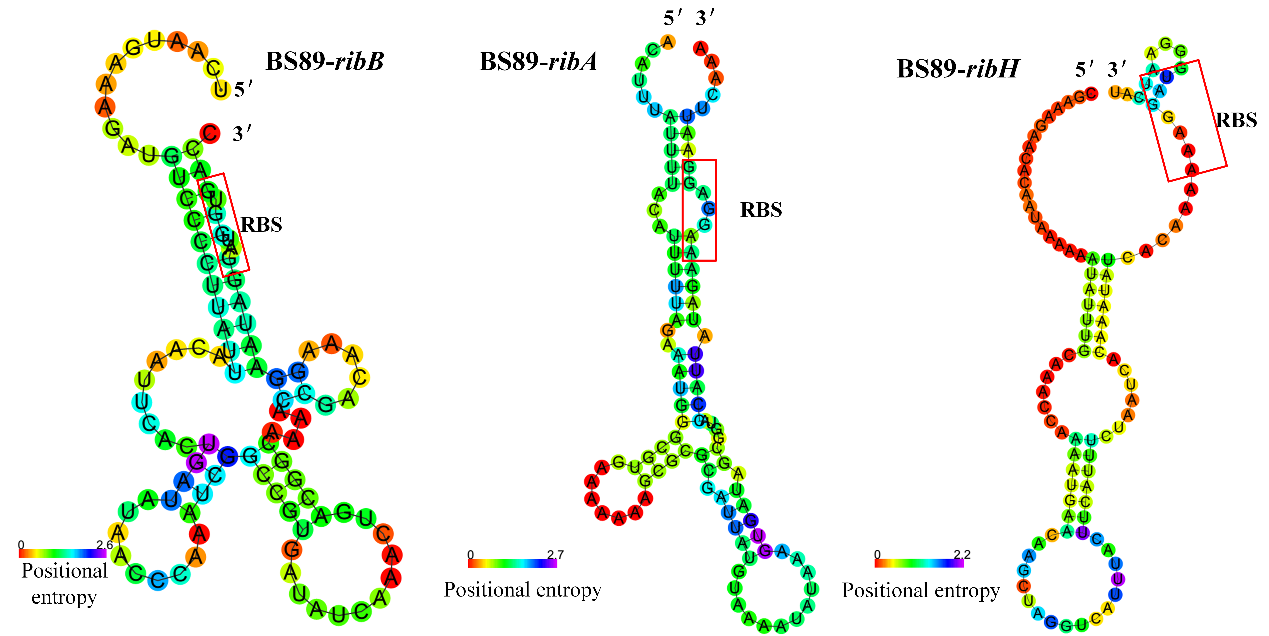
**

**Figure S5.** The secondary structures in the 5’-UTR regions of the mRNA of riboflavin operon genes in strain BS89. The 100 bp upstream sequences of corresponding genes were analyzed using RNAfold software.

**Figure S6**

**
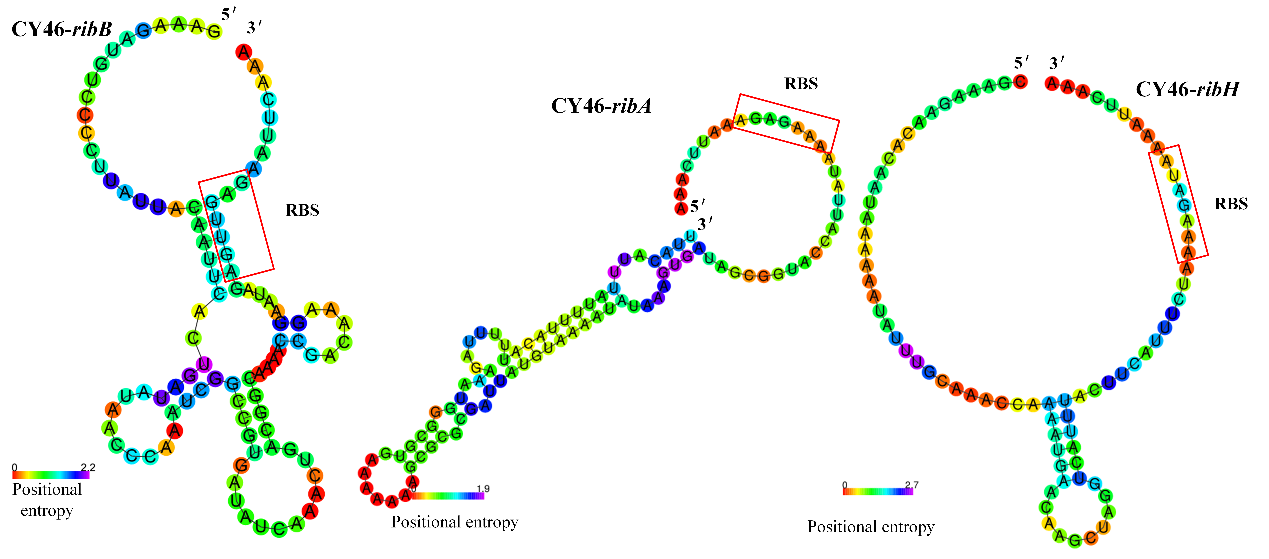
**

**Figure S6.** The secondary structures in the 5’-UTR regions of the mRNA of riboflavin operon genes in strain CY46. The 100 bp upstream sequences of corresponding genes were analyzed using RNAfold software.

**Figure S7**

**
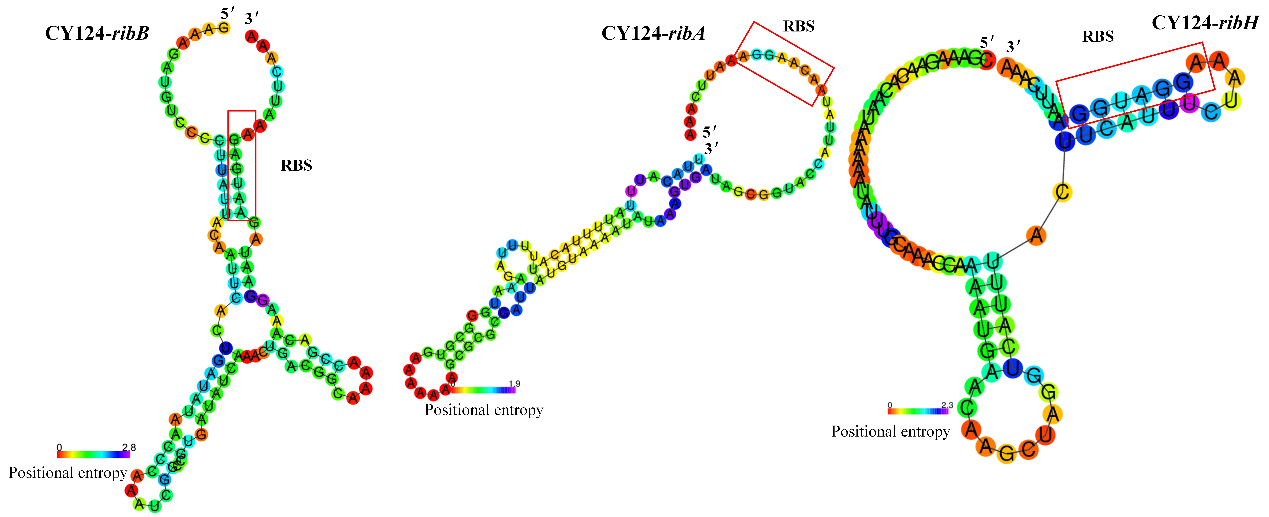
**

**Figure S7.** The secondary structures in the 5’-UTR regions of the mRNA of riboflavin operon genes in strain CY124. The 100 bp upstream sequences of corresponding genes were analyzed using RNAfold software.

**Figure S8**


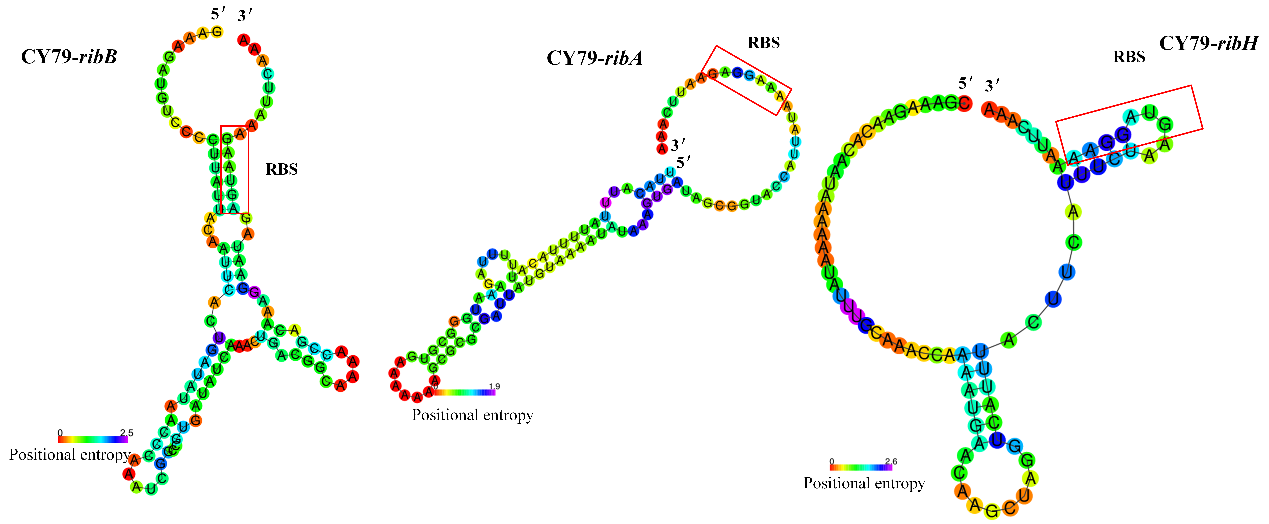


**Figure S8.** The secondary structures in the 5’-UTR regions of the mRNA of riboflavin operon genes in strain CY79. The 100 bp upstream sequences of corresponding genes were analyzed using RNAfold software.

**Figure S9**

**
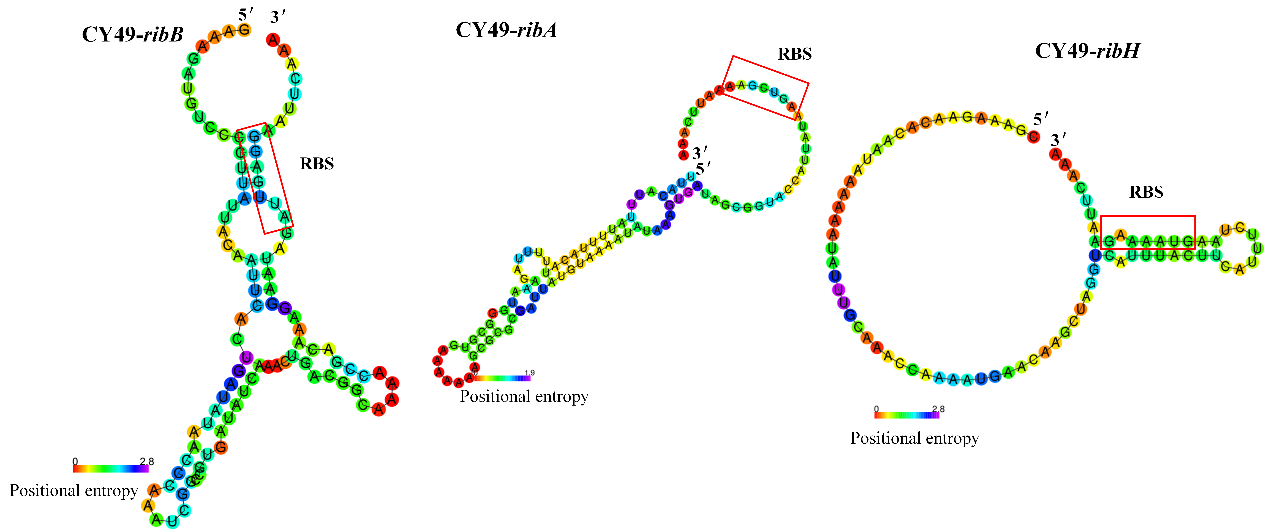
**

**Figure S9.** The secondary structures in the 5’-UTR regions of the mRNA of riboflavin operon genes in strain CY49. The 100 bp upstream sequences of corresponding genes were analyzed using RNAfold software.

**Table S1** Primers and gRNA sequences used in this study

| Primer name | Sequence (5’-3’) | |
| --- | --- | --- |
| P43-F | | CACGCATTGATTTGAGTCAGCTAGGAGGTGACTGACGTGCATGCAGGCCGGGGCAT |
| P43-L | | GTCATCACCGAAACGTGTACATTCCTCTCTTACCT |
| pUC-F | | TAAGGCTAGTCCGTTATCAACTTGAAAAAGTGGCACCCTTCCTCGCTCACTGACTCGC |
| pUC-L | | TTAAAGGATTTGAGCGTAGCGAAAAATCCTTTCTGTCAGACCAAGTTTACTCATAT |
| Cm-F | | CAATCTAAAGTATATATGAGTAAACTTGGTCTGACAGAAAGGATTTTTCGCTACGCTCA |
| Cm-L | | GTGTGATGCGCTGCGTCCATTAAAGGATATGGATCTGGAGCTG |
| *repF*-F | | CAGCTCCAGATCCATATCCTTTAATGGACGCAGCGCATCACAC |
| *repF* -L | | ATTGATTCTTTTTTAAGTGTTACCCCTATAAGTTAGTGTCTTTTGCGCAGTCGGCTTAAAC |
| *xylA*-F | | GTTTAAGCCGACTGCGCAAAAGACACTAACTTATAGGGGTAACACTT |
| *xylA* -L | | TATCTAAGCCTATTGAGTATTTCTTATCCATGGATCCCATTTCCCCCTTTGATTTTTAG |
| Cas9-F | | TCAAAGGGGGAAATGGGATCCATGGATAAGAAATACTCAATAGGCTTAG |
| Cas9-L | | ATGCCCCGGCCTGCATGCACGTCAGTCACCTCCTAGCTGACTCAAATCAAT |
| pDon1-F^a^ | | CACACCA**GGTCTC**AGATTTTAATGAGCTGATTTCGGTATACAGTTGAGACAAGATCTAAGCTTGGCTGCAGGTCGACGGATCCCCGG |
| pDon1-L | | CACACCA**GGTCTC**AAATCGCTTTTTTTACCATAGGTTCCGGTAATAAAGGCATTTTTCCCTGGCGTAATCATGGTCATAGCTGTTTCCT |
| pDon2-F  pDon2-L | | CACACCA**GGTCTC**ATTAAAATAAGGCTAGTCCGTTATCAACTTGAAAAAGTGGCACCAAGCTTGGCTGCAGGTCGACGGATCCCCGG  CACACCA**GGTCTC**ATTAACTTGCTATTTCTAGCTCTAAAACTTACATTTCGAATCGTCAGCAGATCTTGTCTCAACTGTATACCGAAAT |
| D10-F | | TAGGCTTAGCTATCGGCACAAAT |
| D10-L | | TTGAGTATTTCTTATCCATGGATC |
| H840-F | | GATGTCGATGCCATTGTTCCACAAAG |
| H840-L | | ATAATCACTTAAACGATTAATATC |
| Construction of pBSCas9-*amyE*gRNA and pBSCas9n-*amyE*gRNA | | |
|  | | |
| gRNA-*amyE* | | GCGGTGATAGCTTCTCGTTC |
| g*amyE*-F | | CACACCA**GGTCTC**ACTTCTCGTTCGTTTTAGAGCTAGAAATAGCAAGTTAA |
| g*amyE*-L | | CACACCA**GGTCTC**AGAAGCTATCACCGCGTGTACATTCCTCTCTTACCTATAATGGTAC |
| Construction of pBSCas9-Ins1kb*amyE*gRNA and pBSCas9n-Ins1kb*amyE*gRNA | | |
|  | | |
| gRNA-Ins1kb*amyE* | | TAAAAACTGGAAATCAATAA |
| gIns1kb*amyE*-F | | cacacca**ggtctc**aCTGGAAATCAATAAGTTTTAGAGCTAGAAATAGCAAGTTAA |
| gIns1kb*amyE*-L | | cacacca**ggtctc**aCCAGTTTTTAGTACATTCCTCTCTTACCTATAATGGTAC |
| Construction of pBSCas9-Ins2kb*amyE*gRNA and pBSCas9n-Ins2kb *amyE*gRNA | | |
|  | |  |
| gRNA-Ins2kb*amyE* | | GTCAAGAATGTTTGCAAAAC |
| gIns2kb*amyE*-F | | cacacca**ggtctc**aGTTTGCAAAACGTTTTAGAGCTAGAAATAGCAAGTTAA |
| gIns2kb*amyE*-L | | cacacca**ggtctc**aAAACATTCTTGACGTGTACATTCCTCTCTTACCTATAATGGTAC |
| Construction of pBSCas9-*amyE*/*upp*gRNA and pBSCas9n-*amyE*/*upp*gRNA | | |
|  | | |
| gRNA-*amyE* | | GCGGTGATAGCTTCTCGTTC |
| gRNA-*upp* | | GCTGATTCCTGCGGCAAAAG |
| DM1-F | | cacacca**ggtctc**aGCTTCTCGTTCGTTTTAGAGCTAGAAATAGCAAGTTAA |
| DM1-L | | cacacca**ggtctc**aCCGCAGGAATCAGCGTGTACATTCCTCTCTTACCTATAATGGTAC |
| DM2-F | | cacacca**ggtctc**aGCGGCAAAAGGTTTTAGAGCTAGAAATAGCAAGTTAA |
| DM2-L | | cacacca**ggtctc**aAAGCTATCACCGCGTGTACATTCCTCTCTTACCTATAATGGTAC |
| Construction of pBSCas9-*amyE*/*upp*/*sigE*gRNA and pBSCas9n-*amyE*/*upp*/*sigE* gRNA | | |
|  | | |
| gRNA-*amyE* | | GCGGTGATAGCTTCTCGTTC |
| gRNA-*upp* | | GCTGATTCCTGCGGCAAAAG |
| gRNA-*sigE* | | GAAGCTCCCAAACGGCGATC |
| TM1-F | | cacacca**ggtctc**aCTCGTTCGTTTTAGAGCTAGAAATAGCAAGTTAA |
| TM1-L | | cacacca**ggtctc**aGCCGTTTGGGAGCTTCGTGTACATTCCTCTCTTACCTATAATGGTAC |
| TM2-F | | cacacca**ggtctc**aGCGGCAAAAGGTTTTAGAGCTAGAAATAGCAAGTTAA |
| TM2-L | | cacacca**ggtctc**aCGAGAAGCTATCACCGCGTGTACATTCCTCTCTTACCTATAATGGTAC |
| TM3-F | | cacacca**ggtctc**aCGGCGATCGTTTTAGAGCTAGAAATAGCAAGTTAA |
| TM3-L | | cacacca**ggtctc**aCCGCAGGAATCAGCGTGTACATTCCTCTCTTACCTATAATGGTAC |
| Construction of pBSCac9-LGgRNA and pBSCac9n-LGgRNA | | |
| gRNA-LargeDNAFrag | | TTAGCCAGATTGAGAAAAGA |
| gLD-F | | cacacca**ggtctc**aGAGAAAAGAGTTTTAGAGCTAGAAATAGCAAGTTAA |
| gLD-L | | cacacca**ggtctc**aTCTCAATCTGGCTAAGTGTACATTCCTCTCTTACCTATAATGGTAC |
| Construction of pDonor-Del1kb | | |
| Del1kbU-F | | gttttacaacgtcgtgaGATTCAAAACCTCTTTACTGCCGTT |
| Del1kbU-L | | CCGTCTCTGGTCCATTATTGATTTCCAGTTTTTAATTTGTGTGT |
| Del1kbD-F | | ACACACAAATTAAAAACTGGAAATCAATAATGGACCAGAGACGG |
| Del1kbD-L | | ACGCCAGGGTTTTCCCAGTCTAGCCTTGCCCTCAATGGGGAAGAG |
| pDonD1-F | | CTCTTCCCCATTGAGGGCAAGGCTAGActgggaaaaccctggcgt |
| pDonD1-L | | AACGGCAGTAAAGAGGTTTTGAATCTCACGACGTTGTAAAAC |
| Construciton of pDonor-Del2kb | | |
|  | | |
| Del2kbU-F | | gttttacaacgtcgtgaAATGCTCATGCCGAGAATAGACAC |
| Del2kbU-L | | GGTTTCTTTCGGTAAGTCCCGGTTTTGCAAACATTCTTGACACTCC |
| Del2kbD-F | | GGAGTGTCAAGAATGTTTGCAAAACCGGGACTTACCGAAAGAAACC |
| Del2kbD-L | | ACGCCAGGGTTTTCCCAGACTAACGATGCCTTTGAAAATCTTC |
| pDonD2-F | | GAAGATTTTCAAAGGCATCGTTAGTctgggaaaaccctggcgt |
| pDonD2-L | | GTGTCTATTCTCGGCATGAGCATTTCACGACGTTGTAAAAC |
| Construciton of pDonor-Del4kb | | |
|  | | |
| Del4kbU-F | | gttttacaacgtcgtgaGCCGTTTACCGTTCGCCATAAGCTG |
| Del4kbU-L | | CCGATGTACACGTCATCTGCACGCCATGAGAACGCCGGATGTCGGG |
| Del4kbD-F | | CCCGACATCCGGCGTTCTCATGGCGTGCAGATGACGTGTACATCGG |
| Del4kbD-L | | ACGCCAGGGTTTTCCCAGCCGATGTACACGTCATCTGCAC |
| pDonD4-F | | GTGCAGATGACGTGTACATCGGctgggaaaaccctggcgt |
| pDonD4-L | | CAGCTTATGGCGAACGGTAAACGGCTCACGACGTTGTAAAAC |
| Construciton of pDonor-Del6kb | | |
|  | | |
| Del6kbU-F | | gttttacaacgtcgtgaTTCAGGGTTCGCGTGCAGATG |
| Del6kbU-L | | CACCAGCATCGCCGGCCCCTTAAAATCAGAAAGATGATGG |
| Del6kbD-F | | CCATCATCTTTCTGATTTTAAGGGGCCGGCGATGCTGGTG |
| Del6kbD-L | | ACGCCAGGGTTTTCCCAGGAACAGGACTGACAAACGGG |
| pDonD6-F | | CCCGTTTGTCAGTCCTGTTCctgggaaaaccctggcgt |
| pDonD6-L | | CATCTGCACGCGAACCCTGAATCACGACGTTGTAAAAC |
| Construciton of pDonor-Del8kb | | |
|  | | |
| Del8kbU-F | | gttttacaacgtcgtgaGTAAACATCGCATTTCTGTC |
| Del8kbD-L | | CGGGAACACGTTCCCAGATTCCTGAGAAAAACAGCATTGAAATTGCG |
| Del8kbD-F | | CGCAATTTCAATGCTGTTTTTCTCAGGAATCTGGGAACGTGTTCCCG |
| Del8kbD-L | | ACGCCAGGGTTTTCCCAGGTCTACGGCAGCTCGGCGACAAGCGCGG |
| pDonD8-F | | CCGCGCTTGTCGCCGAGCTGCCGTAGACctgggaaaaccctggcgt |
| pDonD8-L | | GACAGAAATGCGATGTTTACTCACGACGTTGTAAAAC |
| Construciton of pDonor-Ins1kb | | |
|  | | |
| Ins1kb-F | | gttttacaacgtcgtgaGATTCAAAACCTCTTTACTGCCGTTATTCG |
| Ins1kb-L | | ACGCCAGGGTTTTCCCAGTCTAGCCTTGCCCTCAATGGGGAAGAG |
| pDonIn1-F | | CTCTTCCCCATTGAGGGCAAGGCTAGActgggaaaaccctggcgt |
| pDonIn1-L | | CGAATAACGGCAGTAAAGAGGTTTTGAATCTCACGACGTTGTAAAAC |
| Construciton of pDonor-Ins2kb | | |
|  | | |
| Ins2kb-F | | gttttacaacgtcgtgaAATGCTCATGCCGAGAATAGACAC |
| Ins2kb-L | | ACGCCAGGGTTTTCCCAGACTAACGATGCCTTTGAAAATCTTC |
| pDonIn2-F | | GAAGATTTTCAAAGGCATCGTTAGTctgggaaaaccctggcgt |
| pDonIn2-L | | GTGTCTATTCTCGGCATGAGCATTTCACGACGTTGTAAAAC |
| Construciton of pDonor-DelLDNA | | |
|  | | |
| DelLDNAU-F | | gttttacaacgtcgtgaTTGATATCGGAGTCAAACAAGACGGGCTTG |
| DelLDNAU-L | | CATATTTTCCCCCACCTAGCTTGCCTAAGCTCTTTGTTATCCATGGTGCAC |
| DelLDNAD-F | | GTGCACCATGGATAACAAAGAGCTTAGGCAAGCTAGGTGGGGGAAAATATG |
| DelLDNAD-L | | ACGCCAGGGTTTTCCCAGCATCTTTTGTTCATAATCAATGCTAG |
| pDonLarDNA-F | | CTAGCATTGATTATGAACAAAAGATGctgggaaaaccctggcgt |
| pDonLarDNA-L | | CAAGCCCGTCTTGTTTGACTCCGATATCAATCACGACGTTGTAAAAC |
| Construciton of pDonor-Mu*amyE* | | |
| Mu*amyE*U-F | | gttttacaacgtcgtgaTCTGATCGATGGGATGTCACGCAGAAT |
| Mu*amyE*U-L | | GAGGCGTACTTTATTAACGAGAAGCTATCACCG |
| Mu*amyE*D-F  Mu*amyE*D-L | | CGGTGATAGCTTCTCGTTAATAAAGTACGCCTC  ACGCCAGGGTTTTCCCAGGATAAACGGCTTTTGTTGTATTCGC |
| pDonMu-F | | GCGAATACAACAAAAGCCGTTTATCctgggaaaaccctggcgt |
| pDonMu-L | | ATTCTGCGTGACATCCCATCGATCAGATCACGACGTTGTAAAAC |
| Construciton of pDonor- Mu*amyE*/*upp* | | |
|  | | |
| DouMu*amyE*U-F | | GTTTTACAACGTCGTGATCTGATCGATGGGATGTCACGCAGAAT |
| DouMu*amyE*U-L | | GAGGCGTACTTTATTAACGAGAAGCTATCACCG |
| DouMu*amyE*D-F | | CGGTGATAGCTTCTCGTTAATAAAGTACGCCTC |
| DouMu*amyE*D-L | | GCTACACGCTGTCTTGCTTCTTCAAGTTGATAAACGGCTTTTGTTGTATTCGC |
| DouMu*upp*U-F | | GCGAATACAACAAAAGCCGTTTATCAACTTGAAGAAGCAAGACAGCGTGTAGC |
| DouMu*upp*U-L | | CATGTCCTTATTATGCCGCAGGAATCAGC |
| DouMu*upp*D-F | | GCTGATTCCTGCGGCATAATAAGGACATG |
| DouMu*upp*D-L | | ACGCCAGGGTTTTCCCAGGCCCCTAACATGTGTCAATCTGTTTAAAG |
| pDonDouMu-F | | CTTTAAACAGATTGACACATGTTAGGGGCctgggaaaaccctggcgt |
| pDonDouMu-L | | ATTCTGCGTGACATCCCATCGATCAGATCACGACGTTGTAAAAC |
| Construciton of pDonor- Mu*amyE*/*upp*/*sigE* | | |
|  | | |
| ThrMu*amyE*U-F | | GTTTTACAACGTCGTGATCTGATCGATGGGATGTCACGCAGAAT |
| ThrMu*amyE*U-L | | GAGGCGTACTTTATTAACGAGAAGCTATCACCG |
| ThMur*amyE*D-F | | CGGTGATAGCTTCTCGTTAATAAAGTACGCCTC |
| ThrMu*amyE*D-L | | GCTACACGCTGTCTTGCTTCTTCAAGTTGATAAACGGCTTTTGTTGTATTCGC |
| ThrMu*upp*U-F | | GCGAATACAACAAAAGCCGTTTATCAACTTGAAGAAGCAAGACAGCGTGTAGC |
| ThrMu*upp*U-L | | CATGTCCTTATTATGCCGCAGGAATCAGC |
| ThrMu*upp*D-F | | GCTGATTCCTGCGGCATAATAAGGACATG |
| ThrMu*upp*D-L | | GCTGTTCGATGGCTTCCAATGGGTCGCCCCTAACATGTGTCAATCTGTTTAAAG |
| ThrMu*sigE*U-F | | CTTTAAACAGATTGACACATGTTAGGGGCGACCCATTGGAAGCCATCGAACAGC |
| ThrMu*sigE*U-L | | GCGCGCTTATTAATCGCCGTTTGGGAGCTTC |
| ThrMu*sigE*D-F | | GAAGCTCCCAAACGGCGATTAATAAGCGCGC |
| ThrMu*sigE*D-L | | ACGCCAGGGTTTTCCCAGGAAATCCCCATCATATCCGCTACATCC |
| pDonThrMu-F | | GGATGTAGCGGATATGATGGGGATTTCctgggaaaaccctggcgt |
| pDonThrMu-L | | ATTCTGCGTGACATCCCATCGATCAGATCACGACGTTGTAAAAC |
| Construciton of pBSCas9n-gRNArib | | |
| gRNA-*ribB* | | AAACCGACAAAGGAATAGGA |
| gRNA-*ribA* | | GATAGCGGTACCATTATAAA |
| gRNA-*ribH* | | AATCACAAATATCACAAAAA |
| grib1-F | | cacacca**ggtctc**aCGACAAAGGAATAGGAGTTTTAGAGCTAGAAATAGCAAGTTAA |
| grib1-L | | cacacca**ggtctc**aTGTGATATTTGTGATTGTGTACATTCCTCTCTTACCTATAATG |
| grib2-F | | cacacca**ggtctc**aCGGTACCATTATAAAGTTTTAGAGCTAGAAATAGCAAGTTAA |
| grib2-L | | cacacca**ggtctc**aGTCGGTTTGTGTACATTCCTCTCTTACCTATAATGGTAC |
| grib3-F | | cacacca**ggtctc**aCACAAAAAGTTTTAGAGCTAGAAATAGCAAGTTAA |
| grib3-L | | cacacca**ggtctc**aACCGCTATCGTGTACATTCCTCTCTTACCTATAATGGTAC |
| Construciton of pDonor-ribRBSLib | | |
| *ribB*U-F | | gttatcaacttgaaaaagtggcaccCAGGATGCTCAGCAATACAGGAAAACACACCAAAGC |
| *ribB*U-L | | CTATTCCTTTGTCGGTTTTGCCGTCAGTTT |
| *ribB*D-F^b^ | | CAAAACCGACAAAGGAATAGRNNNNRRAATTCAAAATGTTTACAGGAATTATCGAAGAA |
| *ribB*D-L | | CTAAAAGCCGTTTTCGCTTAAGAAGGC |
| *ribA*U-F | | GCCTTCTTAAGCGAAAACGGCTTTTAG |
| *ribA*U-L | | TATAATGGTACCGCTATCACTTTATAT |
| *ribA*D-F | | GTGATAGCGGTACCATTATARNNNNRRAATTCAAAATGTTTCATCCGATAGAAGAAGCA |
| *ribA*D-L | | AATGCACCCGGACCAATACCGGTTCTTCTCC |
| *ribH*U-F | | GGAGAAGAACCGGTATTGGTCCGGGTGCATT |
| *ribH*U-L | | TTAGAAATGAAGTAAATGACCTAGCTTGTT |
| *ribH*D-F | | GGTCATTTACTTCATTTCTAARNNNNRRAATTCAAAATGAATATCATACAAGGAAATTT |
| *ribH*D-L | | GACGTTGTAAAACGACGGCCAGTGAAAAAAACCCCTCTATATCCAAAATGTCCAGTCT |
| pDonLib-F | | AGACTGGACATTTTGGATATAGAGGGGTTTTTTTCACTGGCCGTCGTTTTACAACGTC |
| pDonLib-L | | GCTTTGGTGTGTTTTCCTGTATTGCTGAGCATCCTGGGTGCCACTTTTTCAAGTTGATAAC |
| P1-F | | GCAGACTGCCGAATGTAACAAAACAGCCGGTT |
| P1-L | | TTCTCTTCAATTCGTGTGATTTCCGCAGTTCCGTC |
| P2-F | | TGCCGCCGGGGCTGTTTGCGTTTTTGCC |
| P2-L | | CACGATGGTCTATGCTTACGGTAAATGCAGT |
| P3-F | | GGCGCTTGGATTCTTGCCGGATCTTCG |
| P3-L | | GGCAGAAACAGCACAATCTACACCTTTGTTGCCC |

^a^ Restriction enzyme sites are shown in bold.

^b^ Degenerate oligonucleotide N indicates a 25% possibility of each of the A, G, C, and T bases. Degenerate oligonucleotide R indicates a 50% possibility of each of the A and G bases.
